# Designing and running an advanced Bioinformatics and genome analyses course in Tunisia

**DOI:** 10.1371/journal.pcbi.1006373

**Published:** 2019-01-28

**Authors:** Fatma Z. Guerfali, Dhafer Laouini, Abdellatif Boudabous, Fredj Tekaia

**Affiliations:** 1 Université Tunis El Manar, Tunis, Tunisia; 2 Institut Pasteur de Tunis, LR11IPT02, Laboratory of Transmission, Control and Immunobiology of Infections (LTCII), Tunis-Belvédère, Tunisia; 3 Université Tunis El Manar, Faculté des Sciences de Tunis, Laboratoire Microorganisme et Biomolécules Actives, Campus Universitaire Farhat Heched, El Manar, Tunis, Tunisia; 4 Institut Pasteur Paris, 28 rue du Dr Roux, 75724 Paris cedex 15, France; Genome Quebec, CANADA

## Abstract

Genome data, with underlying new knowledge, are accumulating at exponential rate thanks to ever-improving sequencing technologies and the parallel development of dedicated efficient Bioinformatics methods and tools. Advanced Education in Bioinformatics and Genome Analyses is to a large extent not accessible to students in developing countries where endeavors to set up Bioinformatics courses concern most often only basic levels. Here, we report a pioneering pilot experience concerning the design and implementation, from scratch, of a three-months advanced and extensive course in Bioinformatics and Genome Analyses in the Institut Pasteur de Tunis. Most significantly the outcome of the course was upgrading the participants’ skills in Bioinformatics and Genome Analyses to recognized international standards. Here we detail the different steps involved in the implementation of this course as well as the topics covered in the program. The description of this pilot experience might be helpful for the implementation of other similar educational projects, notably in developing countries, aiming to go beyond basics and providing young researchers with high-level skills.

## Introduction

Whereas in developed countries education and training in Bioinformatics and Genome Analyses are now fully integrated in academic curricula, the situation is different in developing countries. Some countries embarked on these domains with the first sequencing projects [1–5], whereas in African countries, apart from some leading Institutions [6–9], the importance of these domains was only later realized [10–11]. A recent program [12] allowed developing many educational opportunities to learn Bioinformatics and Genome Analyses but mainly at the introductory levels [10,13]. In this context, frameworks have been designed and implemented for assessing the capacity of research groups to perform widely used analyses of high-throughput genomic data [14] that have affected genomics and Bioinformatics research in Africa [15].

Taking into account this educational drawback in Tunisia and building on existing efforts and on our experience in working in appropriate environments and in organizing short courses in different countries [16], we volunteered to set up at the Institut Pasteur de Tunis, Tunisia, an advanced three months course in Bioinformatics and Genome Analyses [17] starting from scratch, targeting young researchers and post docs. The challenge was to design and run a program to upgrade the skills in Bioinformatics and Genome Analyses of few selected candidates. Our objective was to go beyond basic and introductory presentations and offer a robust training model. We aimed to familiarize participants with the Unix environment and programming languages (we chose Perl and R) to work in the context of genome data. We considered a three months course so that the participants had enough time to immerse in the methods, projects, data and related literature aspects of large-scale genome analyses.

An important specificity of our course was the weekly Lab meeting day that allowed the participants to follow up the scientific literature relevant to the course topics and train to prepare, present and discuss scientific topics.

In the following we describe how we brought participants into this course to the appropriate skills in Bioinformatics and Genome Analyses allowing them to pursue their research and advanced studies in these domains.

### Local context and course planning

Bioinformatics skills have become prerequisite in many fields in biology and medicine, owed in part to the continuing biological data accumulation and the complexity and scale of questions now being addressed through bioinformatics using genome data [18]. Genomics has proved its numerous applications in many domains such as human health, environment, biotechnology, agriculture, and of course in advancing basic science. In Tunisia, a professional Master in Bioinformatics was launched in 2002 but the program was brought to an end in 2006, due to the lack of funding. Since then bioinformatics and genome science are still not part of the regular cursus of Tunisian universities, no matter the concerned departments (Biology, Computer Sciences, Statistics nor in Applied mathematics).

Many attempts were undertaken to organize short bioinformatics courses of basic or advanced levels, but unfortunately such efforts remained isolated, with little impact on the evolution of the situation. The rising generations are consequently leaving the University with Master and PhD degrees deprived of strong Bioinformatics and Genomics skills, particularly in life sciences including Biology. Such qualified students leave the University with no significant awareness about the new gained knowledge accumulated since the beginning of genome sequencing projects.

In such context, based on previous experience in organizing short advanced courses in “Bioinformatics and Genome Analyses”, mainly financed by EMBO [16], we set up a full three months’ advanced and intensive course devoted to these topics. Although extremely dense and long compared to other courses, this extended length was considered appropriate to solve the specific lack of competences explained above.

We considered several key principles in designing a course program for such targeted participants, which we learned through insights gained across many years of combined experience in developing short courses and from our own experience in analyzing genome data.

Our main objective aimed at delivering an advanced and intensive course in Bioinformatics and Genome Analyses. The specific goals were to familiarize participants with Bioinformatics Methods and Tools used in large-scale genome analyses and with working in a scientific environment i.e. follow up of the scientific literature relevant to the course topics, working on bibliographic projects as well as the preparation and presentation of talks on specific topics.

For such an intensive course we targeted participants with diverse backgrounds (Mathematics, Statistics, Computer science and/or Biology) deliberately deciding to start from scratch. For this purpose, we planned to upgrade the participant’s skills in: a) Unix (one week); b) Perl (one week) and c) methods and tools dedicated to sequence analysis (two weeks).

Subsequently we planned a seven weeks period dedicated to genome analyses (theoretical and practical sessions), detailing methods and tools used in complete genome studies, in Next Generation Sequencing (NGS) and in metagenomics data analyses.

In this overall organization a major focus was put on practical sessions with three quarters of the time devoted to hands-on sessions, and theoretical presentations in the remaining quarter. Finally and in order to train participants to also gain skills in the preparation and publicly presentation of scientific works, we planned a weekly one-day lab meeting to discuss projects and follow up on bibliographic resources. The three months course was based on seven and half hours of work per day, five days a week. The course languages were French and English, depending on the speaker, and all course material and documents were in English.

### Selection of participants

An international public announcement of the course was published along with the planned program, in a dedicated web page [17] and BIOSCI/Bionet e-mail lists (Computational-biology, Bionews). Applicants had to complete a concise questionnaire about their background, their actual situation, their research projects and their motivations to participate to the course (See S1 Text). The rather demanding conditions of the course were explicitly mentioned in the application form.

Evaluation criteria were mainly based on 1) the background which needed to be relevant to the course topics 2) the involvement in research projects and 3) clear expressed motivations, in line with the course topics. Evaluation of received applications was performed by colleagues, already participating in the organization of previous short courses. The evaluators were well aware of the extensive work required from the selected participants during the course.

The twenty top ranked applicants emerging from the evaluation received a “recommendation document”, stating in detail the heavy workload and personal commitment expected from them. The document also emphasized the fact that the course was not a scholar one and that active participation would be needed all along the course.

### Implementation of the course program

The majority of the selected participants had a biology background (with only two in computer science and statistics backgrounds). Unfortunately, for this first set up we could not manage to have a multidisciplinary group as we expected, rendering our project more challenging. We very much regretted the absence in the selected group of equivalent numbers of mathematicians, statisticians and computer scientists as compared to biologists. It is worth mentioning that the large majority (78%) of the participants were female, reflecting a current gender imbalance in many Tunisian Universities.

**Fig 1:**
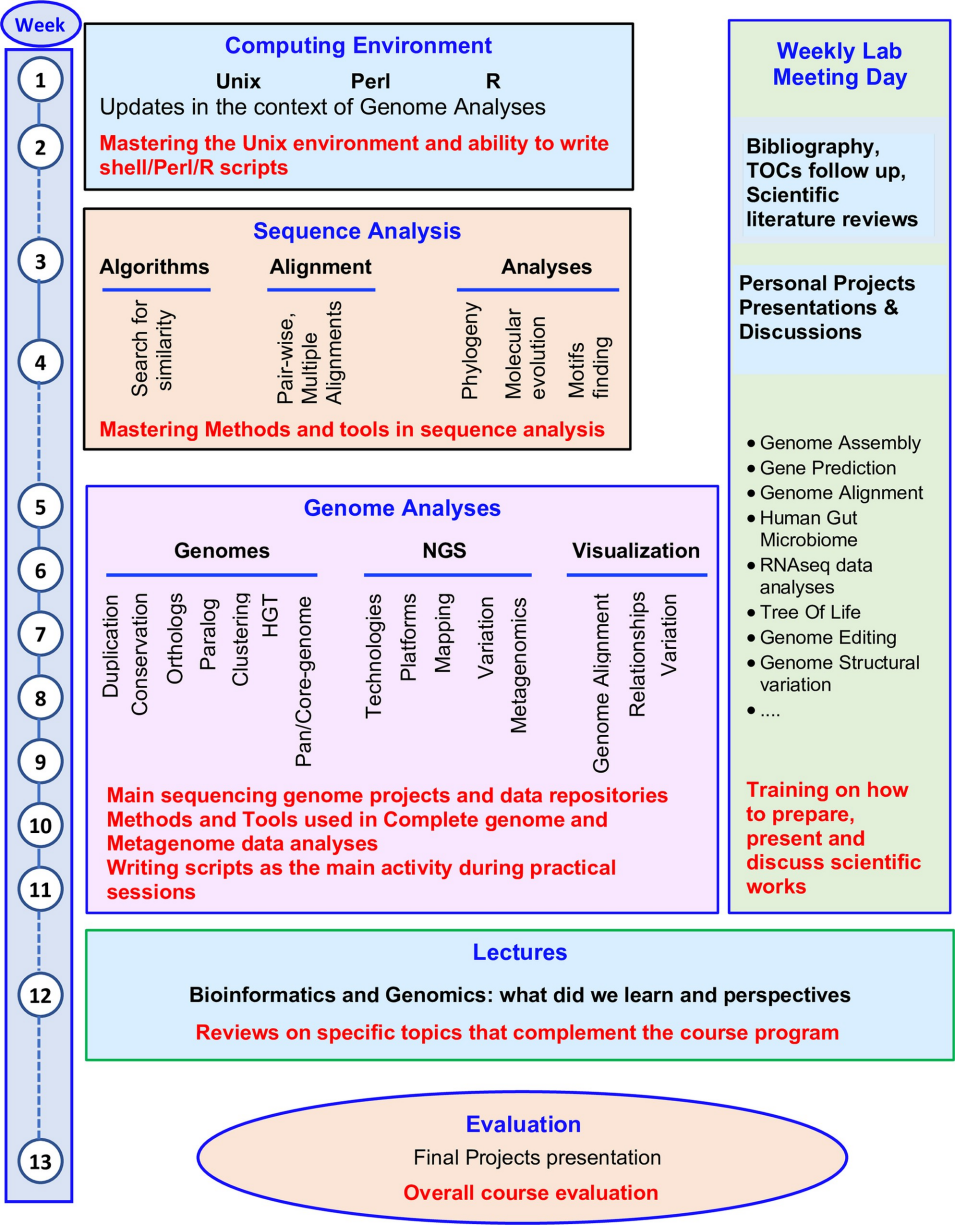
Development of the Bioinformatics and Genome Analyses course. The diagram shows the weekly development of the main topics and activities during the course period. At the left side, the order and number of weeks correspond to the blocs of working topics represented at the right side of the Figure: a) Computing Environment, b) Sequence Analysis, c) Genome Analyses, d) Lectures on Genomics and Bioinformatics, e) Weekly Lab meetings all along the course period and f) the course evaluation.

The program started from scratch. Fig 1 shows the weekly development of the main topics (details and corresponding material are publicly available: [19] and S2 Text). Before the starting of the course, computers were set up to run under Linux operating system with the necessary Unix utilities for the practical sessions. In addition, software needed for the practical sessions were installed.

The course material is available for download from this link:

https://webext.pasteur.fr/tekaia/BCGAIPT2017/BCGAIPT2017_Prog.html

### Unix sh shell and Perl

Apart from remote access through browsers to important web sites (e.g. the ncbi website: https://www.ncbi.nlm.nih.gov), we deliberately avoided to locally work with any menu-driven utilities based on clicking to analyze data. Indeed, in this case, the participants will never grasp what is really going on in making any analysis program work, particularly in terms of parameters set up.

We chose to implement the course in a Unix(Linux) environment. Indeed, Unix shell systems provide access to many specialized utilities that can be executed by online commands [18]. Above all, Unix pipe operator, ‘‘|”, makes it possible to create *ad hoc* pipelines by connecting the output of one command to the input of the next one.

On the other hand, scripting languages, such as Perl and R, are appropriate and effective in solving many biological questions using significant amounts of sequence data or the outputs of diverse analyses, and accordingly several sessions covering both programming languages were included in the program.

Our course program started with one week devoted to skills upgrading in Unix. The hierarchical structure of Unix was introduced, along with most used shell commands paying continuously particular attention during the practical sessions to the end use in terms of genomic analyses.

We considered for all practical sessions, three yeast genomes (*Saccharomyces cerevisiae*, *Candida glabrata* and *Zygosaccharomyces rouxii*) [20] and five mycobacterium genomes (*Mycobacterium tuberculosis* H37R, *Mycobacterium bovis*, *Mycobacterium leprea*, *Mycobacterium marinum* and *Mycobacterium ulcerans*) [21] (complete genome sequences, with their corresponding predicted coding and proteins sequences) (see S3 Text). The choice of these genomes was motivated by their respective abundant scientific literature related to their genome analyses and on their realistic data size, not too small nor too large, allowing a fair understanding of large-scale analyses. Practical sessions involved the manipulation of all these different datasets.

At the end of the first week the participants were able to write simple *sh* scripts implying the use of *grep* and *sed*, concerning notably the search for subsets of protein sequences and the use of simple commands (as for example identification and listing of sequence features, counting, substitution, …) (see details in S4 Text).

The second week was devoted to skills upgrading in Perl programming. Participants were introduced to Perl, detailing its abilities for substitution, selection, reading and writing files. Data types were among the most important concepts the participants had to grasp. The iterative way in dealing with data was part of the many simple scripts that were written as practical exercises (see details in S5 Text).

At the end of the first two weeks, participants were able to master most of the useful Unix commands and also to write simple shell and Perl scripts to solve simple problems.

### Methods and tools in sequence analysis

The next two weeks were devoted to skills upgrading in sequence analysis, introducing key concepts in Biology, in Bioinformatics and in Genome science. More precisely, methods and tools were introduced for pair-wise sequence comparisons, multiple sequence alignments, construction of phylogenies, molecular evolution and motifs finding. These introductions were followed by intensive practical sessions using locally installed programs as well as web resources (see S2 Text and corresponding pdf documents for practical sessions).

Examples of considered hands-on included, (i) gene and protein sequence composition respectively in base and amino-acids, (ii) practical procedure of the Smith-Waterman algorithm in aligning simple sequences, (iii) pairwise local and global sequence (nucleotide and protein) alignments (see S6 Text), (iv) running BLAST programs and interpreting their outputs (see S7 Text), (v) Multiple sequence alignments (ClustalW, ClustalOmega, MAFFT) (see S8 Text), (vi) Phylogenic tree construction methods, using essentially the PHYLIP package (see S9 Text), as well as (vii) aligning nucleotide sequences following their corresponding aligned proteins, particularly useful to ensure alignment at the codons level (see S10 Text). Such alignment outputs were used as basis for exercises on calculation of synonymous and non-synonymous substitutions using PAML [22]. All such practical examples were first run interactively, using command line instructions, and subsequently with automatic procedures relying on somewhat advanced *Perl* and shell scripts written by the participants.

At this step participants were endowed with the required background in Bioinformatics methods and tools used in sequence analysis, in a Unix environment, to start large-scale genome analyses.

### Large-scale genome analyses

This topic represented the heart of the course program. Fundamental methods and tools used in Bioinformatics were introduced along with their effective application in real-case analyses concerning complete genomes and NGS or metagenomics data.

Two weeks were devoted to large-scale analyses of completely sequenced Eukaryotic genomes. Lectures on genome resources, introducing notably major genome projects and associated published results, were followed by practical sessions in large-scale genome analyses, notably with studies of the main evolutionary processes related to intra-species duplication, conservation between species, horizontal gene transfer, inference of paralogs and orthologues and their classification [23,24]. Such practical sessions were based on real data (three yeast genomes), allowing participants to manipulate large amounts of data by writing Perl and sh scripts in a Linux environment. The analyses concerned, notably, the characterization of genomes in terms of size, base compositions and amino-acid compositions of the corresponding proteomes (see details in S11 Text). In addition, the following topics were also considered: the comparison of genomes and determination of degree of intra-species duplications and of inter-species conservations, the determination of families of intra-species paralogs and of pair-wise Reciprocal Best Hits between protein sequences in distinct species and their clustering into families of orthologs using the *mcl* program [25] (see details in S12 Text and S13 Text). Results of intra-species and inter-species comparisons were further displayed using *CIRCOS* [26]. Practical sessions were also devoted to the search for repeats, in the three yeast genomes, using the Tandem Repeat Finder (*trf*) program [27] (see details in S14 Text).

Two more weeks were devoted to the introduction of Genome and Transcriptome studies using NGS technologies as well as an introduction to R and *Rstudio* [28] basics for NGS data analyses. Subsequently, NGS technologies, mapping and analyses, algorithms for read mapping and tandem repeat variants in Human genome were introduced followed by intensive practical sessions.

The following week was devoted to metagenomics data analyses. The steps involved in such analyses were introduced by lectures and applications including, (i) an introduction to microbial ecology and omics; (ii) an introduction to *Tidyverse* [29]; (iii) handling of amplicon sequences; (iv) analyses automation with *make*; (v) Ecological diversity measurements and ordination; and (vi) shotgun methods for environment genomics and introduction to *Vegan* [30].

These steps included data pre-processing (Primer trimming, Quality filtering, Chimera removal); OTU picking (*de novo* or reference-based clustering; Taxonomic assignment); Phylogeny reconstruction (*de novo* or reference-based multiple alignments; Phylogenetic analysis) and data post-processing and analysis (rarefaction curve; alpha-diversity and beta-diversity calculations; feature selection and correlation analysis (see definition in [31]). Intensive practical sessions followed each of these topics.

Finally, the last week of the genomic sessions was devoted to completely sequenced bacterial genomes. Practical sessions considered five completely sequenced *Mycobacterium* genomes for comparisons (pair-wise all versus all) to look for duplication, conservation, paralog, orthologues inference and clustering using *mcl* [25].

The specific aim of these sessions was the mastering of automation solutions, based on the re-use of previously written scripts for yeast genome analyses, to perform all pair-wise comparisons, Reciprocal Best Hits (rbh) determination and clustering, search for motifs in clusters of orthologs using the *meme* [32] program, as well as for genes subject to positive selection using the PAML [22] program (see S15 Text).

### Weekly lab meetings

The second main objective of the course was to improve skills in preparing and presenting talks. The objective was to train participants in discussing scientific topics throughout the scientific literature as well as in preparing scientific documents and publicly presenting talks [33]. For this purpose, a weekly Lab meeting day was organized to discuss relevant papers that were published that week, following Table Of Contents (TOCs) (see S2 Text for references of analyzed publications during each of the Lab meetings), as well as progress in individual bibliographic projects.

During the first Lab meeting a selection of scientific journals was suggested, and participants were asked to “sign in” each of them to receive their corresponding TOCs and present during the Lab meetings some selected papers related to the course topics. Moreover, participants were asked to choose one project among a list of suggested ones aiming at synthesizing the corresponding scientific literature. The project progress was followed during the Lab meetings and a final presentation of each project was scheduled for the end of the course. Examples of such projects include: “Genome assembly methods”, “Genome alignments: algorithmic aspects”, “Gene prediction methods” and “Human gut microbiome as diagnostic marker of diseases” (see the complete list in [19] and in S2 text).

The Lab meetings proved interesting for almost all of the participants as they enriched each other with what they learned in their respective projects and by the published literature they were sharing.

### Final week: Lectures

The course was concluded by a week including a series of lectures entitled “Bioinformatics and Genome studies: what did we learn and perspectives”. The lectures complemented the course practical sessions with experts introducing knowledge recently gained from genomes, NGS and metagenomics studies.

The lecturers included topics such as: “The rise of Genomes and Bioinformatics”, “Complexities of parasite genomes for high-throughput data interpretation”, “Exploring Genome Data using Correspondence Analysis”, “SNV and SV calling”, “RNA-seq”, “Understanding non-coding DNA and data sharing”, “The evolution of the tuberculosis agent”, “Virulence determinants of *Mycobacterium tuberculosis* with a special focus on ESX/type secretion systems”, “Bacterial genomics: from sequencing one genome to thousands of genomes”, “Studying bacterial communities by genomic methods” and “The saga of giant viruses: historical, epistemological and biological aspects”.

In addition, to widen the horizon of the course, two structure-oriented lectures (“Introduction to Crystallography” and “The Impact of structural genomics”) were presented.

All lecture documents are available for download from reference [19].

### Last week: Course evaluation and perspectives

At the end of the cursus a specific session was organized for the evaluation of the course by all participants: students and organizers.

### a) Course evaluation by the participants

Each participant had to evaluate the three months course by completing a questionnaire prepared for this purpose (see S16 Text).

The detailed outcome of the evaluations was in-depth analyzed (see S17 Text for detailed distribution of the evaluations related to each question). Overall the course was highly rated by the students. It appeared that most of the participants were proud to be among the first to benefit from this course. They mentioned positive comments about the international character of the course particularly having speakers from different countries and the high level of the organization and program topics and few negative comments about the heavy workload and efforts they had to make.

Complementary topics have been suggested to be introduced in future course organization of this kind, including in particular theory and practice of methods and tools used in genome assembly, of structural variation analyses (in Human, Plants…) and structural Bioinformatics. More lectures were also suggested to be programmed to the developing Genome Project-Write [34–35] and to synthetic Biology projects [36] as well as to the Microbiota and Microbiome projects and their application in human health and disease.

It is interesting to note that all participants suggested the continuation of organizing such course program in the future.

### b) Participants evaluation by the speakers

Five speakers that participated by at least one week lectures and practical sessions have evaluated at the end of their respective sessions, each participant on a scale of 1 (not enough appropriate for the topic) to 5 (very good) that reflected the awareness and mastering of the delivered topics as well as active participation during their corresponding lectures and practical sessions (commenting, asking questions, suggesting solutions…).

Mean scores were calculated for each participant. The obtained histogram (see S18 Text) shows the distribution of the mean scores (x-axis) following the number of participants (y-axis). The majority of the participants were scored “good” or “very good”.

### c) Lessons and perspectives

Despite the stringent conditions in pursuing this course, it was rewarding to see the student’s enthusiasm in learning and practicing advanced topics they never suspected to be able to experience before, given the huge gap in their university education. Some negative organizational aspects should nevertheless be mentioned and avoided in future.

It is worth mentioning that nothing in this course organization and scientific program was peculiar to Tunisia and international standards were voluntarily followed. We applied the same rules and considered similar conditions as we did in our past short courses [16] organized in many different countries, except its longer period. A prerequisite to such an organization was the availability of a computers’ room and a good connection to the Internet. At Institut Pasteur de Tunis, such conditions were fairly met.

With this experience we are convinced that it is possible to run an advanced course with participants that had almost no experience in Bioinformatics and Genomes, but that were motivated to invest hard efforts to learn. In our opinion, the length of the course period was a fundamental criteria that should be considered to help participants immerse in this studious atmosphere.

Specific to this course program was the inclusion of a weekly day devoted to scientific discussions, follow up of scientific literature and progress in personal bibliographic projects. Indeed, it is important that participants have to be adequately trained to prepare documents, give talks (form and content) and be part of scientific argumentation exchanges. For the majority of the participants but for two participants (see S17 Text), it was a profitable experience, in spite of the implied supplementary workload.

Although improving skills in preparation and presentation of talks should be pursued in future courses, the way to do it should be probably more directive. Projects could be proposed along with a short list of accompanying fundamental published papers related to each topic. This will help participants avoid time searching for adequate literature at the beginning of their project.

It is to be regretted that the course budget was short and therefore had dramatic limitations on the organization. We could not implement some important topics related to genome analyses as for example methods related to genome assembly, genome annotation, structural genome variant analyses, microbiome and human health or system biology topics among others.

Another negative aspect was the small number (20) of available computers. Local efforts should be made to limit this drawback and allow at least thirty participants to benefit from this kind and other courses. In addition, affordable housing possibilities and financial support for potential international participants should also be considered in future organizations.

Finally, a follow-up of the effective involvement of all participants into projects and publications related to bioinformatics and genome analyses would be possible through a once-a-year mailing-survey to ensure that the final objectives of the course are fully achieved.

### Conclusion

Here, we report on achieving the implementation of an extensive Bioinformatics and Genome Analyses course in Tunisia, at advanced level. As the course was the first of its kind in this regional context, it was challenging to design it from scratch. Most importantly, despite their basic starting level in the course topics, it appeared possible to infuse self-confidence to the students, raising up most of them at the end of the course to international standards levels. In this background we believe that the innovative field-experience reported here could be inspirational for the implementation of similar courses in other developing countries, and possibly also in developed countries not providing as yet such educational programs.

It is indeed obvious that there is a need in Tunisia, and in other developing countries for a new generation of educated and well trained Bioinformaticians and genomics specialists, not only for the technical use but also for research developments.

Moreover, in many biological fields of research using large-scale genomic datasets and information, there is a great variety of biological questions that need multidisciplinary competences and approaches to be addressed. In developing countries there is an urgent need to implement a strategy to encourage close collaborative efforts between computational scientists, mathematicians, statisticians and biologists to take part in the research and discovery initiatives in post-genome era.

Additionally, young researchers should also re-think their understanding of Computer Biology: “Computational biologists are just biologists using a different tool” as stated in [37]. It is possible to acquire programming skills that will make young researchers in developing countries, better able to implement, interpret and understand their own analyses, making by the way themselves at the same time better experimentalist as well [37].

Many challenges lie ahead. Awareness is still lacking from mathematicians, statisticians and computer scientists in developing countries and particularly in Tunisia, about the huge opportunities offered by the publicly available data from genome sequencing projects to develop new ideas and take part in this extraordinary discovery adventure. It is their responsibility to make computational biology and genomics known as fields of research on their own. They are warmly invited to consider with attention the Singapore activities in these domains [4] as an example of what a small country can achieve.

We hope this kind of achievement will encourage the Tunisian High Education and Scientific Research administrations to effectively act in favor of the development of these domains and the international community to contribute and help the set-up of such initiatives in developing countries.

## Supporting information

S1 Text Course announcement and application form. This document includes the course announcement and the questionnaire that has been designed for the selection of the participants and that has been completed by applicants to the course.
(DOCX)

S2 Text Detailed course program with references to lectures and hands-on documents. This document includes the detailed course program and relevant references to data, lectures, practical sessions as well as Lab meeting contents.
(DOCX)

S3 Text Data coding convention for practical sessions (pdf). Coding conventions for sequences, species, genomes, proteomes and scripts.
(PDF)

S4 Text Hands-on for the Unix practical sessions (pdf). (PDF)

S5 Text Hands-on for Perl practical sessions (pdf). (PDF)

S6 Text Practical sessions for Sequence comparisons (pdf). (PDF)

S7 Text Practical sessions for Blast programs use and Databases settings (pdf). (PDF)

S8 Text Practical sessions for Multiple Sequence Alignment (pdf). (PDF)

S9 Text Practical sessions for Phylogeny analyses (pdf). (PDF)

S10 Text Practical sessions for Molecular evolutionary analyses (pdf). (PDF)

S11 Text Practical sessions on examples of characterization of some complete genomes (pdf). (PDF)

S12 Text Practical sessions for Large-scale genome comparisons (pdf). (PDF)

S13 Text Practical sessions for Large-scale genome comparisons and Paralogs, Orthologs inference and clustering (pdf). (PDF)

S14 Text Practical sessions for Tandem repeated motifs search (pdf). (PDF)

S15 Text Practical sessions for Paralogs, Orthologs inference and clustering in 5 mycobacterial proteomes (pdf). (PDF)

S16 Text Evaluation questionnaire. This document includes the questionnaire that has been completed by each participant at the end of the course to evaluate the different steps of the course development and realization.
The questionnaire ends with offering the inclusion of free comments.
(DOCX)

S17 Text Statistical results of the completed evaluation questionnaire. This document includes the statistical outputs represented by histograms as obtained from the completed questionnaire (see S16 Text) by all participants. The histograms show the distribution of the evaluations related to each question of the questionnaire (x-axis) following the number of participants (y-axis). On top of each histogram is indicated the corresponding question.
The histograms are followed by the list of mentioned free comments.
(DOCX)

S18 Text Overall evaluation of the participants by the speakers. This document includes the statistical output represented by a histogram, of the participants evaluation scores by 5 speakers who participated by at least one week lectures and practical sessions. The histogram shows the distribution of the obtained mean-scores (x-axis) following the number of participants (y-axis).
The detailed course program and related lectures, documents for practical sessions are also available for public access through the link: https://webext.pasteur.fr/tekaia/BCGAIPT2017/BCGAIPT2017_Prog.html and the GitHub platform: https://github.com/tekaia/BCGAIPT2017.
(DOCX)
