# Supplementary material for: Designing and running an advanced Bioinformatics and genome analyses course in Tunisia
Source: PLoS Comput Biol. 2019 Jan 28;15(1):e1006373. doi: 10.1371/journal.pcbi.1006373 (PMC6349305; doi:10.1371/journal.pcbi.1006373)
Supplement: S1 Text — This document includes the course announcement and the questionnaire that has been designed for the selection of the participants and that has been completed by applicants to the course. (DOCX) [file pcbi.1006373.s001.docx]

**S1 Text: Course announcement and application form**

This document includes the course announcement and the questionnaire that has been designed for the selection of the participants and that has been completed by applicants to the course.

**Bioinformatics and Genome Analysis Course**

**September 18 – December 15, 2017**

**Institut Pasteur Tunis, Tunisia**

**Course web page :** [**https://webext.pasteur.fr/tekaia/BCGAIPT2017.html**](https://webext.pasteur.fr/tekaia/BCGAIPT2017.html)

**• Presentation**

As of today there are about 90000 public genome sequencing projects, in complete of draft states (see recent statistics in <http://www.genomesonline.org/)>, giving rise yearly to thousands of multidisciplinary scientific publications, involving mainly Biologists, Computer Scientists, Statisticians and Mathematicians.

Genomics has applications in Human health (individual genomes, personalized medicine, gene therapy, microbiomes,…), in Biotechnology (energy, food processing,…), in Environment (metagenomics, soil, waters, polluents, oceans,…), in Agronomy (genome editing, population screening, animal and plant selection,…) and in Fundamental research sciences (genome structure, organisation, evolution,...).

This course aims at updating academic students/researchers with Bioinformatics methods and tools used in large-scale Genome analyses, as well as in scientific knowledge so far acquired from genome studies.

The course programme plans to cover the following topics:

• ) Preparation of the participants to work in the Unix(Linux) environment in the context of genome analyses.

•) Sequence analysis methods

•) Genome analyses

•) Series of lecturers

Bioinformatics and Genomes : what did we learn and perspectives.

•) Course evaluation

See up-to-date program at this link:

<https://webext.pasteur.fr/tekaia/BCGAIPT2017/BCGAIPT2017_Prog.pdf>

The main objectives of this Practical Course are to strengthen skills of students in genomics and bioinformatics on the use of algorithms, key software and visualization methods and their various applications in genome studies.
The course program is designed to provide a solid theory class followed by practical sessions on each topic. Students will obtain the dexterity to manipulate large data sets in a Unix environment and a basic understanding of scripting and programming skills.

# • Who can apply

The course is aimed at motivated PhD, candidate PhDs and young researchers from academic Institutions, with backgrounds in a Biology, Mathematics or Computing disciplines. The course is an advanced course intended for candidates familiar with sequence analysis and who need to extend their competences to genome analysis.

The course **is not a scolar course**, it is based on personal and group involvements as well as active participations. **Full-time presence is mandatory**.

Acceptance will be subject to a selection process and preference will be given to research scientists involved in Genome analyses projects.

**• Course langages:** English/Français (Course documents are in English).

**• Selection Criteria**

The selection criteria will include the following:

-Must be graduate student, junior or senior researcher;

-Member of a Research Academic Institution;

-Good knowledge in Molecular Biology, Bioinformatics, Sequence analysis, Programming or Practical Computer skills;

-Able to understand, read and write in English and French;

-Participant must be available full-time for the entire duration of the course;

-Priority will be given to candidates whose research activities will immediately benefit from the course as reflected by their motivations;

-Motivated in participating activeley during the course period;

-A gender balance will be applied as far as possible;

-Able to take care during the course period of his/her travel, accommodation and meals charges;

**• Number of places:** Twenty (20) places are available for the course.

**• Fees:** 1000DT (course documents, USB flash drive).

**• Travel, accommodation and meals are at the participant’s own charge.**

**• How to Apply**:

Candidates are requested to carefully complete the present form, save it as a PDF document : « YourFamilyName_BCGAIPT2017_Application.pdf » and send it to [tekaia@pasteur.fr](mailto:tekaia@pasteur.fr). The Subject of your e-mail should be « Application to the Bioinformatics and Genome Analyses Course 2017 – Tunis » no later than September 03, 2017.

Incomplete documents will not be considered for evaluation.

• Deadlines:

-Closing date for application is: September 03, 2017.

-Selected participants will be informed by: September 06, 2017.

**• Participation Certificate**

At the end of the course, participants will be delivered a « Participation certificate » by the University Tunis El Manar and the Institut Pasteur de Tunis.

**• Reference**

This course is similar to past organized courses :

<https://webext.pasteur.fr/tekaia/BCGA_WProgs.html>

Before applying, applicants are advised to revieww past course materials and references.

**Bioinformatics and Genome Analysis Course**

**September 18 – December 15, 2017**

**Institut Pasteur Tunis, Tunisia**

**Course web page :** [**https://webext.pasteur.fr/tekaia/BCGAIPT2017.html**](https://webext.pasteur.fr/tekaia/BCGAIPT2017.html)

**First Name - Family Name**

| Insert Your recent whole Personal Photo here |
| --- |

**• Personnal informations:**

First name:

Family name:

E-mail address:

Cell Phone number:

Sexe: Male/Female

Age:

Present Professional situation (Student/PhD/Ass.Prof/...):

Academic Institution:

Department:

Address:

City/Town:

Country:

**• Your Supervisor/Head of Department information :**

First name – Family name:

e-mail :

Academic Institution :

Department :

Institution :

Postal Address :

**Note :** Your supervisor/head of department is assumed to be informed about this application.

We may contact her/him if needed.

• Indicate your degree(s) completed at current stage:

**• Estimate your expertise in the following (tick X/line):**

| **Skills in** | **None** | **Elementary** | **Advanced** |
| --- | --- | --- | --- |
| Molecular Biology |  |  |  |
| Mathematics |  |  |  |
| Statistics |  |  |  |
| Computer Science |  |  |  |
| Programming |  |  |  |
| Bioinformatics |  |  |  |
| Sequence similarity search |  |  |  |
| Sequence multiple alignment |  |  |  |
| Phylogeny analysis |  |  |  |
| Genomes |  |  |  |
| Excel/Word |  |  |  |
| Unix |  |  |  |
| Perl |  |  |  |
| R |  |  |  |

**• Do you have a personal computer ? Yes No**

**• If Yes, is-it Unix/Linux based ? Yes No**

**• Outline of your current research/educational projects (insert 300 words maximum) :**

• Motivations for taking this course.

**what are the reasons that led you to apply for this particular course?**

(insert 300 words maximum):

**• List of references to your personal 5 best publications (visible through PubMed):**

**• Insert a short CV (1 page maximum):**
