# Supplementary material for: Designing and running an advanced Bioinformatics and genome analyses course in Tunisia
Source: PLoS Comput Biol. 2019 Jan 28;15(1):e1006373. doi: 10.1371/journal.pcbi.1006373 (PMC6349305; doi:10.1371/journal.pcbi.1006373)
Supplement: S2 Text — This document includes the detailed course program and relevant references to data, lectures, practical sessions as well as Lab meeting contents. (DOCX) [file pcbi.1006373.s002.docx]

**S2 Text: Detailed course program with references to lectures and hands-on documents**

This document includes the detailed course program and relevant references to data, lectures, practical sessions as well as Lab meeting contents.

**Bioinformatics and Genome Analyses Course**

Institut Pasteur Tunis, Tunisia

September 18 – December 15, 2017

Final and effective detailed program with links to corresponding materials:

https://webext.pasteur.fr/tekaia/BCGAIPT2017/BCGAIPT2017_Prog.html

The course is programmed for 3 months, 5 days/week (9h – 12h30 - 14 h – 18h).

In principal ¼ of the time is devoted to lecturers and ¾ time for practical sessions using computers in a Unix (Linux) environment. Equivalent ½ time preparation (bibliographic work outside the course hours) will be needed.

The first week the students will be given a significant number of published papers related to Genomics and Bioinformatics topics. A web page (useful links) including a list of web sites that will serve as reference for the participants will be enriched during the course by new suggested sites.

The students will be offered a large number of working projects (see list of suggested topics at the end). Students had to choose one project to work on during the whole period of the course.

Weekly sessions (Friday) will be devoted to Lab meeting where students will discuss their ongoing assigned projects and to the follow up of the week scientific literature. Each student will have to lead at least one Lab meeting.

The course working languages are English and French depending on the speaker. All course documents are in English.

**Programme overview**

**I. Updates in Unix/perl in the context of genome analysis**

**Programming/Unix (Linux)**

**September 18 – September 29 (2 weeks)**

**II. Sequence Analysis**

**October 2 – October 13 (2 weeks)**

**III. Genome Analyses**

**October 16 – December 1 (6 weeks)**

**IV. Lecturers**

**Bioinformatics – Genomics studies: What did we learn and perspectives**

**December 4 – December 8 (1 week)**

**V. Evaluations**

Reports presentation

**December 11 – December 15 (1 week)**

**Program details**

**Data used during the practical sessions**

For the practical sessions, we will consider three Yeast and five Mycobacterium completely sequenced genomes (complete genome, coding and protein sequences).

(See detailed data description in S3 Text).

**September 18:**

Welcome – Course introduction

Students’ self-introduction

Introduction to the Computing Network environment.

**I. Updates in the context of genome analyses**

Length: 2 weeks

The principal goal is to allow students to master the Unix (Linux) environment (sh shell, perl, emacs, …) that is used in the context of genome analyses.

**Preliminaries notes on considered data and scripts coding identifications:**

Practical sessions will consider solely genome sequences (nucleotide/protein).

We will consider sequences and databases in “fasta” format and will systematically use the following coding extensions: “.seq” for complete dna sequences, “.dna” for coding sequence databases, “.pep” for protein sequence databases, “.prt” for protein sequences and “.dna” for dna sequences.

We will consider completely sequenced genomes relative to three yeast species:

*Saccharomyces cerevisiae* (denoted SACE), *Candida glabrata* (denoted CAGL) and *Zyrosachharomyces rou*xii (denoted ZYRO). Their corresponding:

-complete genome sequences will be denoted respectively: GSACE.seq, GCAGL.seq and GZYRO.seq

-complete set of coding sequences will be denoted respectively: GSACE.dna, GCAGL.dna and GZYRO.dna

-complete set of protein sequences will be denoted respectively: GSACE.pep, GCAGL.pep and GZYRO.pep.

Single chromosome sequence will be denoted: chrI.seq, single ORF sequence will denoted for example: YAL068c.dna and corresponding protein sequence YAL068c.prt.

The five Mycobacterial genomes are detailed in S3 Text.

Note: scripts identification should be self-explanatory and use the following extension:

“.scr” for Unix shell script and “.pl” for Perl script.

For example: *countchr.scr* (for counting chromosomes) and *basecomp.pl* (for base composition).

**A. Maths – Stats – Programming**

The goal is to update the students with basic and fundamental statistical and mathematical backgrounds necessary for sequence/genome analyses (some topics might be introduced during corresponding lecturers).

**B. Mastering the Unix environment**

Length: 3 days

**September 19 (cont) - 20 – 21 - 25**

The Unix updates include:

-Fundamentals of the Unix (Linux) OS and shell -- filesystem, access and basic commands; navigation of the filesystem and basic file operations -- reading, writing, merging, copying, moving and setting permissions of files and directories - display file content…

-File manipulations using sed, file redirection and piping, command line execution of various bioinformatics programs;

- Editor (emacs);

- shell scripting.

**Lecture:** Introduction to Unix (Tekaia_BCGAIPT2017_Unix.pptx)

**Practical sessions:** BCGAIPT2017_UnixPS.pdf (S4 Text)

**Friday September 22: Lab meeting**

Program: Bibliographic projects presentation and assignment;

e-mail “sign in” selected journals to receive respective Table Of Contents.

The list of suggested journals include: Sciences, Nature, Nature Genetics, Plos Biology, Plos Computational Biology, Plos Genetic, Bioinformatics, Nucleic Acid Research, PNAS, BioMed Cenral, BMC Genomics, BMC Biology, Human Molecular Genetics, Microbiology and Molecular Biology.

**September 26 – 27 – 28**

**Perl**

• Fundamentals of scripting with Perl, such as scalars, arrays, variable interpolation, operators (mathematics, conditional, logical), file input/output, printing, loops (if-then-else, for, while), list operations, ...; functions/subroutines, hash arrays and regular expressions;

• Running simple Perl programs;

• Variables (scalars, arrays), printing, variable interpolation, string operations

• Mathematical, conditional and logical operators, file input/output, loops (if-then-else, for, while)

• List operations, regular expressions, functions...

1. -How to use Perl commands in scripts and functions
2. -Case study: Automating BLAST
   1. -Creating FASTA file with sequences on which to use BLAST
   2. -Writing a program to call blast programs, finding the best hits
   3. - Case study: Download a genome of a given species from the ncbi server and perform some database preparations ready to be used with BLAST for genome analyses.

**Lecture:** Introduction to perl programming (Tekaia_BCGAIPT2017_perl.pptx)

**Lecture:** Introduction to perl programming - the minimum to know for practice

(Tekaia_BCGAIPT2017_perl_min.pptx)

**Practical sessions:** BCGAIPT2017_PerlPS.pdf (S5 Text)

**September 28 (14:00 – 18:00):**

Short introductions to:

a) Functions (linear, exponential, logarithms, ...);

b) Matrices (sum, product, transposition, inversion, diagonalization, eigen-value, eigen-vector);

c) Usual models: Markov model, Hidden Markov Model (HMM), Monte-Carlo model,... ;

**Friday September 29: Lab meeting**

Project presentations.

From TOCs of the week: discussion and presentation of the Science paper:

*-Schlebusch CM, Malmström H, Günther T, et al. (2017).*

*Southern African ancient genomes estimate modern human divergence to 350,000 to 260,000 years ago. Science. 358(6363):652-655.*

**II. Updates in Sequence Analyses**

Length: 2 weeks

The goal is to allow students to master the basic data and methods used in sequence analyses.

**October 2 - 5**

Note: October 2 (<https://www.nobelprize.org>: starting day for Nobel price awards)

-Genetics and Molecular Biology (1 session)

Introduction to fundamental basics: gene, protein, DNA, RNA, coding RNA, non-coding RNA, sRNA, genetic code, central dogma…. Terms that are routinely used in bioinformatics.

**Lecture:** Introduction to Biology: Basics and Novel key concepts

(Guerfali_BCGAIPT2017_BasicsofBiology.pptx)

-General introduction to the main sequencing projects and to bioinformatics methods and tools used in the context of genome analyses

**Lecture:** Introduction to Bioinformatics and Genomics

(Tekaia_BCGAIPT2017_Introduction2BioinformaticsGenomics.pptx)

**October 04:** Science Webinar: [The rise of whole genome microbial sequencing: a new era for human microbiome analysis](http://view6.workcast.net/AuditoriumAuthenticator.aspx?pak=1919346739229145)

Access to the webinar : [The webinar can be downloaded from this link)](http://ll1.workcast.net/10542/2053292700927279/Media/10542_82757_2017100420052532.mp4)

-Sequence comparisons: Algorithms – applications

Local Alignment, global alignment, identity, similarity, homology,

Similarity scores, Algorithms: Needleman & Wunsch, Smith-Waterman, Heuristics, Substitution matrices, Statistical evaluation of similarity scores,…

**Lecture:** Sequence Comparisons: Search for Similarity

(Tekaia_BCGAIPT2017_SimilaritySearch.pptx)

**Practical sessions**: BCGAIPT2017_SeqCompPS.pdf (S6 Text)

-BLAST introduction and use (Blast programs, substitution matrices, Blast algorithm, Smith-Waterman, Blast formatted databases,…)

**Lecture:** Blast introduction and use

(Tekaia_BCGAIPT2017_BLASTintro_use.pptx)

**Practical sessions**: see BCGAIPT2017_BLASTuse.pdf (S7 Text)

-Search for motifs, protein domains, repeated motifs in sequences (nucleic, proteins) (meme, trf)

**Friday October 6: Lab meeting**

-Projects progress presentations

-CRIPSR-Cas special presentation (definition, applications)

Presentations from TOCs of the week:

-Chari R, Church GM. (2017).

Beyond editing to writing large genomes.

Nat Rev Genet. 18(12):749-760. doi: 10.1038/nrg.2017.59.

-Mensh B, Kording K (2017).

Ten simple rules for structuring papers. PLoS Comput Biol 13(9): e1005619. <https://doi.org/10.1371/journal>. pcbi.1005619

-McIntyre et al. (2017).

Comprehensive benchmarking and ensemble approaches for metagenomics classifiers

Genome Biology. 18:182. DOI 10.1186/s13059-017-1299-7.

**October 9 - 12**

-Introduction to the main journals and bibliographic web resources related to Bioinformatics and Genomes – Scientific work citation and impact – News groups related to Bioinformatics and Genomes.

**Lecture**: Bibliographic resources for genome data analyses.

(Tekaia_BCGAIPT2017_BibResources.pptx)

-Global alignment of at least three sequences, Algorithm, different programs

**Lecture:** Multiple Sequence Alignments

(Tekaia_BCGAIPT2017_MultAlign.pptx)

**Practical sessions:** See BCGAIPT2017_msaPS.pdf (S8 Text)

- Phylogenetic tree construction methods (Parsimony, Distance, Maximum likelihood), example of phylogenetic trees, procedure for phylogenetic tree constructions, phylogenetic tree evaluation, applications.

**Lecture:** Molecular Phylogeny Reconstruction methods in the Genome era

(Tekaia_BCGAIPT2017_Phylogeny.pptx)

**Practical sessions:** See BCGAIPT2017_PhylAnalPS.pdf (S9 Text)

-Molecular evolution: Methods (genetic code, synonymous/non-synonymous substitution, selection pressure) – application using PAML.

Example of cluster of orthologs, codons alignment deduced from corresponding amino-acids alignments, selection pressure,

**Lecture:** Molecular Evolution

(Tekaia_BCGAIPT2017_MolEvol.pptx)

**Practical sessions**: See BCGAIPT2017_MolEvolPS.pdf (S10 Text)

**Friday October 13: Lab meeting**

Projects progress presentations.

Presentations from TOCs of the week:

-Dannemann M, Kelso J. (2017).

The Contribution of Neanderthals to Phenotypic Variation in Modern Humans

Am J Hum Genet. 101(4):578-589.

-Zahn LM (2017). A Fantastic Voyage in Genomics. Science 358 (6359), 56-57.

**III. Genome Analyses**

Length: 6 weeks

The goal is to master genomic data and the methods and tools used in comparing complete genome data as well as NGS data.

**Complete Genomes**

**October 16 - 17**

-Chronology of sequencing, Important landmarks in genomics, Main running genome projects, Resources for complete genome sequences, statistics on genome sizes, NGS and new challenges, Completely sequenced genomes web sites (ncbi, ebi, sanger,...).

Complete genomes: Bacteria – Archaea – Eukaryotes – Viruses

**Lecture:** Genome Sequencing: Main Projects and Resources

(Tekaia_BCGAIPT2017_Genome_Resources.pptx)

**Practical sessions**: See BCGAIPT2017_ExplorGenomResoursPS.pdf

- Exploration of available data (sequencing projects web sites)

**-**Compositions, Description

Base composition, genome size,

GC composition; amino-acids compositions;

Protein size, distribution/proteome;

Count number of sequences per chromosome in GSACE.pep

Split a fasta database into individual sequences.

**Practical sessions**: See BCGAIPT2017_CompleteGenomesPS.pdf (S11 Text)

**October 18 – 19 - 23**

b) Genome comparisons

(Duplication, Conservation, syntheny, introgression, HGT, Orthologs, paralogs, clustering... Core-genome, Pan-genome) (**mcl**).

**Practical session:** writing scripts for genome comparisons; extraction of significant hits; best Reciprocal Hits, all hits, orthologs, paralogs, and their clustering using mcl.

For the obtained clusters of orthologs and paralogs: motifs extraction using MEME; selection pressure (automation of previously written scripts);

**Plan:**

**Intra-species comparisons:**

For each proteome, we will perform the following:

-Compare each proteome to itself, using *blastp* (with adequate options);

-Get for each protein its best significant match (presented in a table form);

-Get for each protein all its significant matches (presented in a table form);

-For each protein, calculate the number of its significant matches (presented in a table form);

-mcl clustering into families of non-unique proteins.

**Interspecies comparisons:**

-Perform all pair-wise proteome comparisons;

**For each pair of proteomes:**

-Get for each protein its best significant hit in each of the cognate proteomes;

-Get for each protein all its significant hits in each of the cognate proteomes;

-For each protein calculate the number of its significant matches (presented in a table form);

**Multiple comparisons:**

-Extract all pairs of proteins that are Reciprocal Best Hits;

-Construct clusters (families) of orthologs using the RBH pairs and mcl;

-Construct a conservation Profile (phylogenetic profile) of each protein;

-Construct a corresponding numerical conservation profile;

**Practical sessions:**

See detailed instructions in BCGAIPT2017_GenomCompPS.pdf

See detailed instructions in BCGAIPT2017_ParalogsOrthologsPS.pdf (S13 Text)

**Friday 20: Lab meeting**

Projects progress presentations.

Presentations from TOCs of the week:

- Shendure J, Balasubramanian S, Church GM, Gilbert W, Rogers J, Schloss JA, Waterston RH. (2017).

DNA sequencing at 40: past, present and future. Nature. 550(7676):345-353.

- Nussinov R, Papin JA. (2017).

How can Computation advance microbiome research?

PLoS Comput Biol. 2017 Sep 21;13(9):e1005547. doi: 10.1371/journal.pcbi.1005547.

- Hager CL, Ghannoum MA. (2017).

The mycobiome: Role in health and disease, and as a potential probiotic target in gastrointestinal disease. Dig Liver Dis. 49(11):1171-1176.

- Good BH, McDonald MJ, Barrick JE, Lenski RE, Desai MM. (2017)

The dynamics of molecular evolution

over 60,000 generations. Nature. 551(7678):45-50.

- Robin van der Lee, Laurens Wiel, Teunis J.P. van Dam and Martijn A. Huynen (2017).

Genome-scale detection of positive selection in nine primates predicts human-virus evolutionary conflicts. Nucleic Acids Res. 45(18):10634-10648.

**October 24**

c) Intra and inter-genomic relationships visualization with CIRCOS.

The presentations and practical sessions suggested in this link were followed:

<http://www.circos.ca/documentation/course/>

**October 25**

d) Phylogenomics (Methods – Applications)

Introduction to Phylogenomy: Species trees, Genome Trees.

Introduction to Horizontal Gene Transfer

(Lecture: Tekaia_BCGAIPT2017_Phylogenomy.pptx)

**Practical sessions:**

Sequences concatenation – multiple alignment - Phylogeny

**NGS data analyses**

**Friday October 27: Lab meeting**

Projects progress presentations.

Presentations from TOCs of the week:

-Cohen Jon (2017). Base editors' open new way to fix mutations. *Science* 358 (6362), 432-433.

- Eggertsson HP, Jonsson H, Kristmundsdottir S, et al. (2017).

Graphtyper enables population-scale genotyping using pangenome graphs.

*Nat Genet*. 2017 Nov;49(11):1654-1660. doi: 10.1038/ng.3964.

- Koskella B, Hall LJ, Metcalf CJE. (2017).

The microbiome beyond the horizon of ecological and evolutionary theory.

*Nat Ecol Evol*. 2017 Nov;1(11):1606-1615. doi: 10.1038/s41559-017-0340-2.

- Teh BT, Lim K, Yong CH, Ng CCY, et al. (2017).

The draft genome of tropical fruit durian (*Durio zibethinus*).

*Nat Genet*. 49(11):1633-1641. doi: 10.1038/ng.3972.

**October 26 – 30 – 31. November 1 - 2**

Introduction to NGS: From theory to data analysis?

- Next-Generation Technologies: General introduction and potential applications

- Next-Generation Technologies: overview of web resources for sequencing platforms

- Overview of the DNA-seq analysis protocol: Library preparation

- Overview of the DNA-seq analysis protocol: file formats

- Quality control

- Reads alignment

- Sequencing coverage

- Detecting variation (SNPs and Indels calling)

- Annotating variation

- Interpreting variation

- Representing variation

**Friday November 3: Lab meeting**

Projects progress presentations.

Presentations from TOCs of the week:

- Donovan H. Parks, Christian Rinke, Maria Chuvochina, et al. (2017).

Recovery of nearly 8,000 metagenome-assembled genomes substantially expands the tree of life. *Nature Microbiology* 2, 1533–1542.

- Andrzej Zielezinski, Susana Vinga, Jonas Almeida and Wojciech M. Karlowski. (2017).

Alignment-free sequence comparison: benefits, applications, and tools.

*Genome Biology* 18:186 DOI 10.1186/s13059-017-1319-7.

-Thompson LR, Sanders JG, McDonald D, et al. (2017). A communal catalogue reveals Earth's multiscale microbial diversity. *Nature*. 551(7681):457-463.

- Kaiser J. (2017). Gut microbes shape response to cancer immunotherapy.

*Science*. 358(6363):573. doi: 10.1126/science.358.6363.573.

-Nasmyth K. (2017). How are DNAs woven into chromosomes?

*Science*. 358(6363):589-590. doi: 10.1126/science.aap8729.

**November 6 – 9.**

-Large-scale genome analyses: practical sessions continuation (mcl clustering of orthologs – meme analyses of clusters of orthologs)

**Practical sessions:** BCGAIPT2017_GenomCompPS.pdf (S12 Text)

**Practical sessions:** BCGAIPT2017_ParalogsOrthologsPS.pdf (S13 Text)

-Introduction to whole genome alignments

(Lecture: Tekaia_BCGAIPT2017_GenomesAlignments.pptx)

-Wrap up.

**Friday November 10: Lab meeting**

Projects progress presentations.

Presentations from TOCs of the week:

- Yamagishi J, Asada M, Hakimi H, Tanaka TQ, Sugimoto C, Kawazu SI. (2017).

Whole-genome assembly of *Babesia ovata* and comparative genomics between closely related pathogens.

*BMC Genomics*. 18(1):832. doi: 10.1186/s12864-017-4230-4.

-Zhang DF, Zhi XY, Zhang J, Paoli GC, Cui Y, Shi C, Shi X. (2017).

Preliminary comparative genomics revealed pathogenic potential and international spread of *Staphylococcus argenteus*.

*BMC Genomics*. 2017 Oct 23;18(1):808. doi: 10.1186/s12864-017-4149-9.

- Melinda A. Yang, Xing Gao, Christoph Theunert, et al. (2017).

40,000-Year-Old Individual from Asia Provides Insight into Early Population Structure in Eurasia. *Current Biology* 27, 3202–3208.

**NGS technologies- Algorithms**

**November 13 – 16.**

-Next generation sequencing technologies -Algorithms for read mapping, pattern detection, and pattern matching
-Tandem repeat genetic variation analysis in the human genome

(Lecture: Benson_BCGAIPT2017_NGS_technology_mapping_structural_variants.pptx

Lecture: Benson_BCGAIPT2017_Read_Mapping_Algorithm.pptx

Lecture: Benson_BCGAIPT2017_TandemRepeat_Variants_In_The_Human_Genome.pptx)

**Practical sessions:** notebook1, notebook2, notebook3, noteboot4.

Practical sessions: introduction: search for tandem repeats using Tandem Repeat Finder (trf) program.

(Lecture: Tekaia_BCGAIPT2018_trf.pptx)

**Practical sessions:** BCGAIPT2017_MotifSearchPS.pdf (S14 Text)

**Friday November 17: Lab meeting**

Projects progress presentations.

Presentations from TOCs of the week:

- Kupferschmidt K. (2017).

Genomes rewrite cholera's global story. Science 358 (6364), 706-707.

- Weill et al., (2017). Genomic history of the seventh pandemic of cholera in Africa.

Science 358, 785–789.

- Domman et al., (2017). Integrated view of *Vibrio cholerae* in the Americas.

Science 358, 789–793.

- Booker et al. (2017). Detecting positive selection in the genome.

BMC Biology. 15:98. DOI 10.1186/s12915-017-0434-y.

- Narzisi G, Mishra B. (2011).

Comparing *De Novo* Genome Assembly: The Long and Short of It.

PLoS ONE 6(4): e19175. doi:10.1371/journal.pone.0019175.

- Sergey Koren, Brian P. Walenz, Konstantin Berlin, et al. (2017).

*Canu*: scalable and accurate long-read assembly via adaptive k-mer weighting and repeat separation. Genome Research. 27:722–736.

**Metagenomics**

**November 20 - 24**

-Metagenomics

- Metagenomics assembly

- Quantitative metagenomics

- Overview of bioinformatics analyses for metagenomics data

(Lecture: Lundin_BCGAIPT2017_Introduction2microbialEcologyAndOMICS.pptx

Lecture: Lundin_BCGAIPT2017_Introduction2Rand_tidyverse.pptx

Lecture: Lundin_BCGAIPT2017_Automation_with_make.pptx

Lecture: Lundin_BCGAIPT2017_Handling_amplicon_sequences.pptx

Lecture: Lundin_BCGAIPT2017_Ecological_diversity_measurements_ordination.pptx

Lecture: Lundin_BCGAIPT2017_Shotgun_methods4environmental_genomix.pptx

Lecture: Lundin_BCGAIPT2017_WorkingWith_vegan.pptx

Lecture: Lundin_BCGAIPT2017_Microbial_Ecology.pptx)

**Practical sessions**

**Friday November 24: Lab meeting**

Projects progress presentations.

Presentations from TOCs of the week:

- Pennisi E. (2017). Survey of archaea in the body reveals other microbial guests.

Science 358 (6366), 983.

**November 27 – 30**

**Complete genomes (Bacteria)**

**Practical sessions:**

-Bacterial genome analyses

Consider 5 mycobacterial completely sequenced genomes (see S3 Text);

Simple description: GC%, aa composition, gene/protein sizes; intra & inter proteome comparisons; Paralogs/orthologs inference and clustering using mcl

Follow the practical work that have been done for the yeast genomes (see BCGAIPT2017_GenomComp_PS.pdf (S12 Text)) and adapt to the five Mycobacterial proteomes.

BCGAIPT2017_ParalogsOrthologsPS.pdf (S13 Text) (adapted for Mycobacterial proteomes).

**Practical sessions:** BCGAIPT2017_ParalogsOrthologsMycoPS.pdf (S15 Text)

**Friday November 30: Lab meeting**

Preparation of the final projects presentations and training on final talks presentation.

**December 4 - 8**

**IV. Lectures: Bioinformatics and Genomes studies: What did we learn and perspectives**

Length: 1 week

The course ended with a series of lectures reviews on specific topics giving insights on what we learned and what are the research and applications perspectives.

It is expected that these lectures (1H30 each) will be helpful for participants who intend to pursue their research carriers in the course topics.

The lecturers was open for some selected colleagues and students.

**December 4**

- The rise of Bioinformatics and Genomes

Fredj Tekaia, Invited Researcher, Institut Pasteur Paris.

(Lecture: Tekaia_BCGAIPT2017_TheRiseOfGenomesAndBioinformatics.pdf)

-Complexities of parasite genomes for high-throughput data interpretation: Leishmania as an example.

Fatma Guerfali. Researcher, Institut Pasteur Tunis, Tunisia.

(Lecture: Guerfali_BCGAIPT2017_ComplexitiesOfParasiteGenomes.pdf)

-Exploring Genome Data Using Correspondence Analysis

Fredj Tekaia, invited Researcher, Institut Pasteur Paris.

(Lecture: Tekaia_BCGAIPT2017_ExploringGenomeDataUsingCA.pdf)

**December 5**

- Genomic Variation (SNPs, Indel)

- Genome Structural variation (CNVs, SVs)

Guillaume Bourque, Associate Professor, Bioinformatics Lab, McGill, U. Montreal, Canada.

(Lecture: Bourque_BCGAIPT2017_SNV_SV.pdf

Lecture: Bourque_BCGAIPT2017_NGS_analysis.pdf)

-The evolution of the tuberculosis agent

-Virulence determinants of *Mycobacterium tuberculosis* - special focus on ESX/type VII secretion systems

Roland Brosch, Professor, Institut Pasteur Paris, France.

(Lecture: Brosch_BCGAIPT2017_EvolutionOfTheTuberculosisAgent.pdf

Lecture: Brosch_BCGAIPT2017_VirulenceDeterminantOfMtb-ESX.pdf)

**December 6**

- Epigenomics (ChIP-seq, RNA-seq)

- Methylation data analysis

Guillaume Bourque, Associate Professor, Bioinformatics Lab, McGill, U. Montreal, Canada.

(Lecture: Bourque_BCGAIPT2017_RNA-seq.pdf

Lecture: Bourque_BCGAIPT2017_Non-codingDNA.pdf)

- Bacterial genomics: from sequencing one genome to thousands of genomes

- Studying bacterial communities by genomic methods: Gut microbiome dynamics and antimicrobial resistance ecology

Philippe Glaser, Professor, Institut Pasteur Paris, France.

(Lecture: Glaser_BCGAIPT2017_BacterialGenomics.pdf

Lecture: Glaser_BCGAIPT2017_GutMicrobiomeDynamics-amrEcology.pdf)

**December 7**

- The Saga of giant viruses: historical, epistemological, and biological aspects

-Fundamental difficulties in the annotation and phylogenetic analysis of giant (and regular) DNA viruses.

Jean-Michel Claverie, Professor, CNRS, Univ. Aix-Marseille, France.

(Lecture: Claverie_BCGAIPT2017_GiantViruses.pdf)

-Selected presentations from participants’ projects (see list below)

**December 8**

- Overview of structural genomics: from structure to function.

- The impact of structural genomics

Pedro Alzari, Professor, Institut Pasteur Paris, France.

(Lecture: Alzari_BCGAIPT2017_Intro2Crystallography.pdf

Lecture: Alzari_BCGAIPT2017_ImpactOfStructuralGenomics.pdf)

-Selected presentations from participants’ projects (see list below)

**V. Course Evaluation**

**December 11 – 15**

Projects presentations and participants’ evaluations (see list below).

**December 13**

Participants: Course evaluation

<https://webext.pasteur.fr/tekaia/BCGAIPT2017/TALKS/BCGAIPT2017_Evaluations.pdf>

<https://webext.pasteur.fr/tekaia/BCGAIPT2017/TALKS/BCGAIPT2017_OralEvaluations.pdf>

(large file)

Participation certificates distribution

Organizers comments and suggestions.

**December 14-15**

Scientific and financial reports draft versions.

**Lab Meetings: List of final projects**

| **F_name** | **L_name** | **Project** |
| --- | --- | --- |
| Bouthaina | Ksibi | Genome assembly methods |
| Hedia | Tnani | Gene prediction methods |
| Bacem | Saada | Genome alignments: algorithmic aspects |
| Ikram | Ben Fraj | Human Microbiome as a Diagnostic Marker for Diseases |
| Naira | Dekhil | *Mycobacterium Tuberculosis*: WGS bottlenecks in the characterization of the resistome |
| Amal | Boukteb | RNAseq data analyses strategies and applications in plant |
| Haifa | Chahed | Tardigrade genome |
| Ghada | Nouairia | Tree Of Life construction: methodological aspects |
| Boutheina | Marnissi | DNA aptamers selection: new approach in diagnosis and therapy |
| Nawel | Trabelsi | Red blood cell disorders |
| Mayssa | Chattaoui | From DNA barcoding to e-DNA meta-barcoding approach |
| Salma | Abbes | Evolutionary Dynamics in *Saccharomycetacea* phylum |
| Alif | Chebbi | Metagenomics in sea environment |
| Imen | Mougou | Genome editing |
| Cherifa | Ayari | Third Generation Sequencing |
| Hafedh | Ben Zaabza | Methods for genomic structural variation analyses |
| Saifeddine | Azouz | NGS data analyses for colorectal cancer diagnosis |

**Suggested plan for final report (presentation)**

-Title

-Abstract (Synthetic presentation)

-Topic introduction

-Review of the topic

-Discussion/Conclusion on the research advances

-Research/Application Perspectives

-Final Bibliographic references

Mensh B, Kording K. (2017) Then Simple rules for structuring papers

PLoS Comput Biol. 13(9):e1005619. doi: 10.1371/journal.pcbi.1005619.
