# Supplementary material for: Designing and running an advanced Bioinformatics and genome analyses course in Tunisia
Source: PLoS Comput Biol. 2019 Jan 28;15(1):e1006373. doi: 10.1371/journal.pcbi.1006373 (PMC6349305; doi:10.1371/journal.pcbi.1006373)
Supplement: S3 Text — Coding conventions for sequences, species, genomes, proteomes and scripts. (PDF) [file pcbi.1006373.s003.pdf]

S3 Text: Data and coding conventions for practical sessions (pdf)

Coding conventions for sequences, species, genomes, proteomes and scripts.

Bioinformatics and Genome Analyses
September 18 – December 15, 2017. Institut Pasteur Tunis
https://webext.pasteur.fr/tekaia/BCGAIPT2017.html

Data (see directory DATA):
For the practical sessions three yeast genomes will be used for the first part of the course and five Mycobacterial genomes for bacterial genome comparisons. We will use few examples of sequences (amino-acids and dna) corresponding to already computed cluster of orthologs (SuperPartition of Orthologs) and are denoted SPOn.m where n is the number of sequences (proteins or genes) and m is an arbitrary order. Such examples include: SPO11.1.pep and SPO11.1.dna.
Other examples will be presented when used.

Sequence and genome files:
We consider sequences and databases in "fasta" format and will systematically consider the following conventions:
DB.pep (extension ".pep" for protein sequence database);
DB.dna (extension ".dna" for coding sequence database);
seq.prt (extension ".prt" for protein sequence);
seq.dna (extension ".dna" for dna sequence);
GSPEC.seq (extension ".seq" for complete genome/chromosome sequence);

We will consider completely sequenced genomes relative to three yeast species: *Saccharomyces cerevisiae* (denoted SACE), *Candida glabrata* (denoted CAGL) and *Zyrosachharomyces rou*xii (denoted ZYRO).
Their corresponding:
-complete genome sequences will be denoted respectively: GSACE.seq, GCAGL.seq and GZYRO.seq
-complete set of coding sequences will be denoted respectively: GSACE.dna, GCAGL.dna and GZYRO.dna
-complete set of protein sequences will be denoted respectively: GSACE.pep, GCAGL.pep and GZYRO.pep.

A single ORF sequence will be denoted SeqIdent.dna or Seqident.prt. For example: YAL068c.dna and corresponding protein sequence YAL068c.prt.

Saccharomyces cerevisiae (SACE): 16 chromosomes

Species	Code	Chromosomes	Size	#genes
Saccharomyces cerevisiae	SACE	A	230218	94
Saccharomyces cerevisiae	SACE	B	813184	406
Saccharomyces cerevisiae	SACE	C	316620	161
Saccharomyces cerevisiae	SACE	D	1531933	754
Saccharomyces cerevisiae	SACE	E	576874	277

Saccharomyces cerevisiae	SACE	F	270161	126
Saccharomyces cerevisiae	SACE	G	1090940	527
Saccharomyces cerevisiae	SACE	H	562643	281
Saccharomyces cerevisiae	SACE	I	439888	207
Saccharomyces cerevisiae	SACE	J	745751	357
Saccharomyces cerevisiae	SACE	K	666816	312
Saccharomyces cerevisiae	SACE	L	1078177	508
Saccharomyces cerevisiae	SACE	M	924431	460
Saccharomyces cerevisiae	SACE	N	784333	393
Saccharomyces cerevisiae	SACE	O	1091291	536
Saccharomyces cerevisiae	SACE	P	948066	464

Candida glabrata (CAGL): 13 chromosomes

Species	Code	Chromosomes	Size	#genes
Candida glabrata	CAGL	A	491328	200
Candida glabrata	CAGL	B	502101	212
Candida glabrata	CAGL	C	558804	230
Candida glabrata	CAGL	D	651701	283
Candida glabrata	CAGL	E	687738	278
Candida glabrata	CAGL	F	927101	383
Candida glabrata	CAGL	G	992211	434
Candida glabrata	CAGL	H	1050361	460
Candida glabrata	CAGL	I	1100349	462
Candida glabrata	CAGL	J	1195132	514
Candida glabrata	CAGL	K	1302831	556
Candida glabrata	CAGL	L	1455689	575
Candida glabrata	CAGL	M	1402899	615

Zygosaccharomyces rouxii (ZYRO) : 7 chromosomes

Species	Code	Chromosomes	Size	#genes
Zygosaccharomyces rouxii	ZYRO	A	1114666	580
Zygosaccharomyces rouxii	ZYRO	B	1388208	706
Zygosaccharomyces rouxii	ZYRO	C	1464093	774
Zygosaccharomyces rouxii	ZYRO	D	1496342	768
Zygosaccharomyces rouxii	ZYRO	E	881646	416
Zygosaccharomyces rouxii	ZYRO	F	1554288	806
Zygosaccharomyces rouxii	ZYRO	G	1865392	941

Total number of predicted proteins per species:

species	Code	Number of predicted proteins
Saccharomyces cerevisiae	SACE	5863
Candida glabrata	CAGL	5203
Zygosaccharomyces rouxii	ZYRO	4991

Corresponding data are located in the DATA/Yeast_data directory.
For the bacterial genome comparisons five Mycobacterial genomes will be considered: *Mycobacterium tuberculosis* H37R (GMYTU.seq, GMYTU.dna, GMYTU.pep), *Mycobacterium bovis* (GMYBO.seq, GMYBO.dna, GMYBO.pep), *Mycobacterium leprea* (GMYLE.seq, GMYLE.dna, GMYLE.pep), *Mycobacterium marinum* (GMYMA.seq, GMYMA.dna, GMYMA.pep) and *Mycobacterium ulcerans* (GMYUL.seq, GMYUL.dna and GMYUL.pep).

Total number of predicted proteins per species:

Species	Code	Number of predicted proteins
M. tuberculosis H37R	MYTU	3996
Mycobacterium bovis AF2122/97	MYBO	3920
Mycobacterium leprae	MYLE	1614
Mycobacterium_marinum	MYMA	5483
Mycobacterium_ulcerans	MYUL	5105

Corresponding data are located in the DATA/MYCOBACT_data directory.

Scripts (see directory SCRIPTS):
During the practical sessions we will write Unix shell and perl scripts. Scripts identification should be self-explanatory and use the following extension:
script.pl (extension ".pl" for perl scripts);
script.scr (extension ".scr" for unix shell scripts);
For example: *countchr.scr* (for counting chromosomes) and *basecomp.pl* (for base composition).

Fredj Tekaia (tekaia@pasteur.fr)
