# Supplementary material for: Designing and running an advanced Bioinformatics and genome analyses course in Tunisia
Source: PLoS Comput Biol. 2019 Jan 28;15(1):e1006373. doi: 10.1371/journal.pcbi.1006373 (PMC6349305; doi:10.1371/journal.pcbi.1006373)
Supplement: S4 Text — (PDF) [file pcbi.1006373.s004.pdf]

S4 Text: Hands-on for the Unix practical sessions (pdf).

Bioinformatics and Genome Analyses
September 18 – December 15, 2017. Institut Pasteur Tunis
https://webext.pasteur.fr/tekaia/BCGAIPT2017.html

- *home-directory, mkdir, cd, pathway, pwd, find, more, wc, sort, | (pipe), grep, cut, sed ;* use « *tab* » as separator;

navigate in a hierarchical structure of directories

ls -l; ls -alt; echo $PATH

Add a new path on your *$PATH*

Update your "*.bashrc"* file

Train on using the text editor: *emacs*.

Working on genome sequences:

• Show the GSACE.seq content:
cat GSACE.seq
more GSACE.seq

• How many chromosome sequences are in GSACE.seq?
grep ">" GSACE.seq | more
grep ">" GSACE.seq | wc -l

• Consider the protein database GSACE.pep.
In this *fasta* formatted database: each protein identification is followed by its corresponding functional annotation (generally the gene name or "hypothetical" if no function is known).
cat GSACE.pep
more GSACE.pep

• Count the number of sequences in GSACE.pep
grep "^>" GSACE.pep | wc -l

• List the sequence identifications in GSACE.pep and direct the output to SACE.ident file
grep "^>" GSACE.pep | sed -e "s/>//g" -e "s/ .//g" > SACE.ident*

• In this database, sequence identifications are subject to a specific coding scheme: The first character is Y (as Yeast), the second is the chromosome number (A,B,C,..., P), then the letter R (right) or L (left), then a number (three characters) indicating the order on the chromosome and the last character: C (Crick) or W (Watson) corresponding to the gene orientation (few adds -A, -B,.. to insert new numbers).

• How many sequences are on chromosome A?

grep “^>YA” GSACE.pep | wc -l

• How many sequences are on chromosome O?
grep “^>YO” GSACE.pep | wc -l

• How many sequences are “hypothetical” proteins?
grep -i “hypothetical” GSACE.pep | wc -l

• How many sequences in chromosome A are annotated as “hypothetical”?
grep “>YA” GSACE.pep | grep -i “hypothetical” | wc -l

• Write a shell script to count the number of sequences per chromosome (in GSACE.pep)
Countseqperchr.scr > SACEseqperchr.tab

• Consider the precomputed data table “allSACESACEhits” corresponding to the BLAST comparison of each SACE protein sequence versus the whole GSACE.pep database (all versus all blast comparison).
The table includes respectively: the query sequence identification, the corresponding hit sequence, the identity score, the query sequence length, the start and end positions of the matching segment as well as the number of gaps. Similar information for the hit sequence (length, start, end positions and number of gaps), the matching segment length, the e-value and similarity score.

• Extract columns 1 and 2?
cut -f 1-2 allSACESACEhits > temp

•Extract column 1 and sort the output results?
cut -f 1 allSACESACEhits | sort > allquerysorted
Note: Later we will calculate from this file the multiple matches corresponding to each identification.

Organization and hints:
During the course we will use programs like blast, clustalw, phylip, PAML,…, data like blast formatted databases and write shell and perl scripts.
In order to make easy use of these programs and scripts and to access these data we need to pay attention to adequately parametrize some system parameters and files.

We will consider the following convention, staring from your home directory:
• ~/home0/gensoft/ will include a directory per program (package).
For example: ~/home0/gensoft/blast/
~/home0/gensoft/phylip/
~/home0/gensoft/PAML/
~/home0/data/
For example: ~/home0/data/GSACE.pep
~/home0/data/ will include the blast formatted databases as well as directories for individual sequences related to a given species. Exp. allsaceprt.fasta

Create a directory in your home directory: ~/bin/
This directory will include your tested scripts i.e. when you make sure the scripts you write run correctly.
the *~/bin* directory should be on your $PATH.

~/test_directories
You consider many directories where you will perform your tests

We will have to progressively update the *$PATH* parameter and the ".bashrc" file.
For example when the blast programs are copied in the ~/home0/gensoft/blast/ directory, we will change the $PATH as follows in the ".bashrc" file:
$PATH=$PATH:~/home0/gensoft/blast/
and execute the on-line command "source .bashrc".

Some programs need an explicit initiation as for example for the blast program that need to know where the blast formatted databases reside:
Export $BLASTDB=~/home0/data/BLASTDB/
such command should be inserted in your ".baschrc" file.

We will have to update such parameters and others during the course.

Fredj Tekaia (tekaia@pasteur.fr)
