# Supplementary material for: Designing and running an advanced Bioinformatics and genome analyses course in Tunisia
Source: PLoS Comput Biol. 2019 Jan 28;15(1):e1006373. doi: 10.1371/journal.pcbi.1006373 (PMC6349305; doi:10.1371/journal.pcbi.1006373)
Supplement: S5 Text — (PDF) [file pcbi.1006373.s005.pdf]

S5 Text: Hands-on for Perl practical sessions (pdf).

Bioinformatics and Genome Analyses
September 18 – December 15, 2017. Institut Pasteur Tunis
https://webext.pasteur.fr/tekaia/BCGAIPT2017.html

References:
Programming Perl (Book by Larry Wall) 3rd edition
https://docstore.mik.ua/orelly/perl/prog3/index.htm

Perl Tutorial:
https://www.tutorialspoint.com/perl/index.htm

Common Practices (Programming Perl):
https://docstore.mik.ua/orelly/perl/prog3/ch24_01.htm

Sample Perl scripts (examples):
http://sifaka.cs.uiuc.edu/czhai/learnperl.html

Practical session:

I. Consider simple sequences (dna or amino acids (aa))
Example: YAL068c.dna and YAL068c.prt

•Write a script to calculate:
-base composition; GC% for nucleotide sequences

•Write a script to calculate:
Amino-acid composition for protein sequences

-sequence size (base or amino-acids);

-codon counts

-extract a segment from a sequence corresponding to start and position;

Extractpartseq.pl (sequence_identification, start_position, end_position)

II. Use "goldData.xls" to compute different statistics about genome sequencing projects.
goldData.xls was downloaded from: https://gold.jgi.doe.gov/downloadexceldata
in august 2017. Most recent data can be downloaded from this URL.

-Save the file goldData.xls into a text formatted file: goldData.txt with <tab> as column separator.

-Write scripts to transform "\r" into "\n" in goldData.txt:
trcr2nl.pl goldData.txt &

output file is: goldData.txt.nl
mv goldData.txt.nl goldData.txt

-Replace white space " " in goldData.txt by "_" and Extract subsets of columns that will be used for next steps and keep in the output file solely the following columns:

GOLDSTAMP PROJECT_TYPE PROJECT_STATUS SEQUENCING_STATUS DOMAIN PHYLUM SPECIES
(corresponding to columns: 0, 5, 6, 7, 12, 14, 19).
extracttabgenomes.pl
The output file is goldDatafinal.txt

-Extract from goldDatafinal.txt, data tables each corresponding to a Domain (create a table per domain: Bacteria/Archaea/Eukaryotes/Viruses): output should be: Bateria.tab, Archaea.tab, Eukaryotes.tab, Viruses.tab.
extractDomain.pl &

-Statistics: *stats.pl &*
The script *stats.pl* performs the following steps:

For each domain extract the following data table:
-WGS_Complete output file: Domain_WGS_Complete.tab
-WGS_Incomplete output file: Domain_WGS_Incomplete.tab
-WGS_Permanent_Draft output file: Domain_WGS_Permanent_Draft.tab
-Transcriptome_Complete output file: Domain_Transcriptome_Complete.tab
-Transcriptome incomplete output file: Domain_Transcriptome_incomplete.tab
and corresponding number of elements (size of the table).

Note "Domain" is Archaea/Bacteria/Eukaryote/Viruses.

The corresponding statistics output file is: *statisticsgoldData.txt*.

-Compute the PHYLUM distributions in each of the above output files related to a given domain.

Distributions of phylum per Project type:
WGS Complete
Bacteria: Bacteria_Complete.freq
Archaea: Archaea_Complete.freq
Eukaryotes: Eukaryotes_Complete.freq

WGS incomplete
Bacteria: Bacteria_Incomplete.freq
Archaea: Archaea_Incomplete.freq
Eukaryotes: Eukaryotes_Incomplete.freq

WGS Permanent draft
Bacteria: Bacteria_Permanent_Draft.freq

Archaea: Archaea_Incomplete.freq
Eukaryotes: Eukaryotes_Permanent_Draft.freq

Transcriptome Incomplete
Eukaryotes: Eukaryotes_Transcriptome_Incomplete.freq

For example: "PHYLUM" (column 6) distribution (in completely sequenced genomes).
From each of these tables (Domain_WGS_Complete.tab) compute the
Phylum distribution/Domain (number of occurrences/per phylum in decreasing order)

Phylum distribution in Bacteria:
cut -f 6 Bacteria_WGS_Complete.tab | sort > ph

Write a script (freqsortednames.pl) that computes occurrences/identification.
freqsortednames.pl ph & #(output file: ph.freq)
Note: eliminate "Phylum" from ph

sort -n -k 2 -r ph.freq > Bacteria_Complete.freq
(insert a column header : « Phylum Nb » at the first line).

Phylum distribution in Archaea:
cut -f 6 Archaea_WGS_Complete.tab | sort > ph
freqsortednames.pl ph &
Note: eliminate "Phylum" from ph

sort -n -k 2 -r ph.freq > Archaea_Complete.freq
(insert a column header : « Phylum Nb » at the first line).

and so on for Eukaryotes and Viruses.

For example: Eukaryotes_Complete.freq
Phylum Nb
Ascomycota 163
Streptophyta 50
Chordata 34
Apicomplexa 19
None 15
Nematoda 11
Arthropoda 10
Platyhelminthes 6
Basidiomycota 6
Microsporidia 5
Mucoromycota 2
Chlorophyta 2
Eustigmatophyceae 1
Bacillariophyta 1

III. Data manipulation:

Consider the GFF file (to be download from the ncbi server) :
GCF_000018785.1_ASM1878v1_genomic.gff

-What is the GFF format (search using google – look at the ncbi FAQ,..)?

od –c GCF_000018785.1_ASM1878v1_genomic.gff | more

can you guess the structure of this file (how many columns/line)?

Fredj Tekaia (tekaia@pasteur.fr)
