# Supplementary material for: Designing and running an advanced Bioinformatics and genome analyses course in Tunisia
Source: PLoS Comput Biol. 2019 Jan 28;15(1):e1006373. doi: 10.1371/journal.pcbi.1006373 (PMC6349305; doi:10.1371/journal.pcbi.1006373)
Supplement: S6 Text — (PDF) [file pcbi.1006373.s006.pdf]

S6 Text: Practical sessions for Sequence comparisons (pdf).

Bioinformatics and Genome Analyses
September 18 – December 15, 2017. Institut Pasteur Tunis
https://webext.pasteur.fr/tekaia/BCGAIPT2017.html

Explore pair-wise local and global sequence alignments (see ebi server https://www.ebi.ac.uk/Tools and fasta server: https://fasta.bioch.virginia.edu/fasta_www2/fasta_list2.shtml) and save the output files with appropriate identifications.
Consider 2 protein sequences: KLTH0D17710g.prt and KLTH0G02794g.prt and 2 nucleotide sequences: KLTH0D17710g.dna and KLTH0G02794g.dna

Local alignment
https://www.ebi.ac.uk/Tools/psa/emboss_water/
Water uses the Smith-Waterman algorithm to calculate the local alignment of two sequences (protein or dna)

Nucleotide sequences:
https://www.ebi.ac.uk/Tools/psa/emboss_water/nucleotide.html

LALIGN
Two protein sequences
https://www.ebi.ac.uk/Tools/psa/lalign/

Two nucleotide sequences
https://www.ebi.ac.uk/Tools/psa/lalign/nucleotide.html

Consider using the FASTA server:
https://fasta.bioch.virginia.edu/fasta_www2/fasta_list2.shtml

LALIGN finds non-overlapping local alignments (protein or dna sequences)
https://fasta.bioch.virginia.edu/fasta_www2/fasta_www.cgi?rm=lalign&pgm=lal
Interesting to explore: *PLALIGN* (plot protein:protein sequences or DNA:DNA sequences)

Blast 2 sequences
allows to get local alignments of 2 sequences:
blastp –query Seq1.prt –subject Seq2.prt –out Seq2sequences.blp

Global alignment
• Consider 2 protein sequences: KLTH0D17710g.prt and KLTH0G02794g.prt
Use this URL to get their global alignment
https://www.ebi.ac.uk/Tools/psa/emboss_needle/

• Consider 2 nucleotide sequences: KLTH0D17710g.dna and KLTH0G02794g.dna
Use this URL to get their global alignment
https://www.ebi.ac.uk/Tools/psa/emboss_needle/nucleotide.html

• Blast Global Alignment: Needleman-Wunsch Global Align Protein/Nucleotide Sequences (ncbi server):
https://blast.ncbi.nlm.nih.gov/Blast.cgi?PAGE_TYPE=BlastSearch&PROG_DEF=blastn&BLAST_PROG_DEF=blastn&BLAST_SPEC=GlobalAln&LINK_LOC=BlastHomeLink

Explore the dot matrix view (dotplot) and the detailed global alignment.

Fredj Tekaia (tekaia@pasteur.fr)
