# Supplementary material for: Designing and running an advanced Bioinformatics and genome analyses course in Tunisia
Source: PLoS Comput Biol. 2019 Jan 28;15(1):e1006373. doi: 10.1371/journal.pcbi.1006373 (PMC6349305; doi:10.1371/journal.pcbi.1006373)
Supplement: S7 Text — (PDF) [file pcbi.1006373.s007.pdf]

S7 Text: Practical sessions for Blast programs use and Databases settings (pdf).

Bioinformatics and Genome Analyses
September 18 – December 15, 2017. Institut Pasteur Tunis
https://webext.pasteur.fr/tekaia/BCGAIPT2017.html

Use of the ncbi server:
We will first use the Blast programs on the ncbi server *https://blast.ncbi.nlm.nih.gov/Blast.cgi* then we will use them locally.

Using two sequences YAL067c.prt and YAL067c.dna, run all combination of programs that are offered on the ncbi site then use the locally installed *blastp* and *blastn* to compare them versus GSACE.pep and GSACE.dna databases (see below).

Local use:
The directory where the reside the blast programs should be added to the $PATH variable (update your ".bashrc" file with the adequate $PATH).
Similarly update the $BLASTDB parameter with the proper path to the directory where the Blast formatted databases are installed.
We will move the blast programs to the directory: ~/home0/gensoft/blast/ and the blast formatted databases to the ~/home0/data/ directory.
The following two lines should be added to ".bashrc" file:
export $BLASTDB=~/home0/data/
$PATH=$PATH: ~/home0/gensoft/blast/

• Formatting databases (GSACE.pep and GSACE.dna) for use with the Blast programs:
makeblastdb –in GSACE.pep –dbtype prot –title "S. cerevisiae proteome"
makeblastdb –in GSACE.dna –dbtype nucl –title "S. cerevisiae genes"

Using blastp

The essential options are:
-*db* subject database (created with *makeblastdb*)
-*query* input sequence file (query sequence in FASTA format)
-*out* output file name

Examples of basic syntax with *blastp* and blastn, on the command line:
blastp –query YAL063c.prt –db GSACE.pep –out YAL063c.blp
blastp –query YAL063c.prt –db GSACE.pep -outfmt 6 –out YAL063c.blp6
blastp –query YAL063c.prt –db GSACE.pep -html –out YAL063c_blp.html
blastp –query YAL067c.prt –db GSACE.pep –outfmt 6 –out YAL067c.blp6
blastp –query YAL067c.prt –db GSACE.pep –matrix BLOSUM80 –out YAL067c.blp
blastp –query YAL067c.prt –db GSACE.pep –seg yes –out YAL067c.blpseg
blastp -query YAL067c.prt -db GSACE.pep -evalue 1.e-9 -out YAL067c.blp-9
blastp -query YAL067c.prt -db GSACE.pep -html -out YAL067c.blp.html

blastn -query YAL067c.dna -db GSACE.dna -out YAL067c.bln

• Analyze the different obtained outputs.

• Blast 2 sequences (comparing two protein sequences):
blastp –query Seq1.prt –subject Seq2.prt –out Seq2sequences.blp

Running BLAST programs with Perl:
The most commonly used Perl command for running external programs is "*system*". This command executes the program specified by its arguments, then returns control to the next line in your Perl program.
The simplest way to use "*system*" is to simply enclose the command line you need in quotes:
system("blastp –query QuerySeq –db Database –out outputfile");

Fredj Tekaia (tekaia@pasteur.fr)
