# Supplementary material for: Designing and running an advanced Bioinformatics and genome analyses course in Tunisia
Source: PLoS Comput Biol. 2019 Jan 28;15(1):e1006373. doi: 10.1371/journal.pcbi.1006373 (PMC6349305; doi:10.1371/journal.pcbi.1006373)
Supplement: S8 Text — (PDF) [file pcbi.1006373.s008.pdf]

S8 Text: Practical sessions for Multiple Sequence Alignment (pdf).

Bioinformatics and Genome Analyses
September 18 – December 15, 2017. Institut Pasteur Tunis
https://webext.pasteur.fr/tekaia/BCGAIPT2017.html

Multiple alignment

• Use the Clustal Omega program: *https://www.ebi.ac.uk/Tools/msa/clustalo/*
to align members of the clusters of orthologs: SPO11.1.pep and SPO11.1.dna and save results into appropriate file. (see more examples of clusters of orthologs).
Preferable output format: clustalw (later PHYLIP)
Explore some parameters (percent identity matrix, show color,…)

• Use the MAFFT (Multiple Alignment using Fast Fourier Transform) program:
https://www.ebi.ac.uk/Tools/msa/mafft/ to align the same members of the clusters of orthologs SPO11.1.pep and SPO11.1.dna and save results into appropriate file.

Preferable output format: clustalw.
Explore some parameters (percent identity matrix, show color,..).

• You may explore other multiple sequence alignment program on this server:
https://www.ebi.ac.uk/Tools/msa/

Using clustalw locally:

• Use *clustalw* to align protein sequences included in a cluster of orthologs SPO11.1.pep and redirect the default output *clustal* formatted alignment to SPO11.1.pep.aln
clustalw –infile=SPO11.1.pep –align –outfile=SPO11.1.pep.aln 2>&1 > /dev/null &

• Use *clustalw* to align protein sequences included in a cluster of orthologs SPO11.1.pep and redirect the output *PIR* formatted alignment to SPO11.1.pep.pir
clustalw –infile=SPO11.1.pep –align –output=PIR –outfile=SPO11.1.pep.pir 2>&1 > /dev/null &

• Use the same file and redirect the output *PHYLIP* formatted alignment to SPO11.1.pep.phy
clustalw –infile=SPO11.1.pep –align –output=PHYLIP –outfile=SPO11.1.pep.phy 2>&1 > /dev/null &

• Do the same for SPO11.1.dna
clustalw –infile=SPO11.1.dna –align –outfile=SPO11.1.dna.aln 2>&1 > /dev/null &
clustalw –infile=SPO11.1.dna –align –output=PIR –outfile=SPO11.1.dna.pir 2>&1 > /dev/null &
clustalw –infile=SPO11.1.dna –align –output=PHYLIP –outfile=SPO11.1.dna.phy 2>&1 > /dev/null &

• Compare all above obtained multiple alignments for each of SPO11.1.pep and SPO11.1.dna

• Write a shell script *clust.scr* that runs *clustalw* with input file and alignment format as input variables

```
#!/bin/sh
#usage: clust.scr filename output_format
clustalw –infile=$1  –align  –output=$2   –outfile=$1.$2  2>&1 > /dev/null
```

• Write a shell script clustall.scr that uses the clust.scr for a set of clusters of orthologs (with extension ".pep")

```
#!/bin/sh
#usage: clustall.scr output-format
for file in `ls *.pep`
do
clust.scr $file $1
done
```

Fredj Tekaia (tekaia@pasteur.fr)

• Write similar scripts using Perl
-Write a *Perl* script *clust.pl* that runs *clustalw* with input file and alignment format as input variables

```
#!/usr/bin/perl
#usage: clust.pl filename output_format
$IN1=@ARGV[0];
$IN2=@ARGV[1];
system (”clustalw –infile=$IN1  –align  –output=$IN2   –outfile=$IN1.$IN2  2>&1 > /dev/null”);
```

-Write a *Perl* script *clustall.pl* that uses the *clust.pl* for a set of clusters of orthologs (with extension ".pep")

```
#!/usr/bin/perl
#usage: clustall.pl OF (output format)
$of=@ARGV[0]; # output format
@list=`ls *.pep`;
while ($F=shift(@list))
{
system("clust.pl $F  $of");
}
```

• Try *Gblock* to show colored alignment
http://molevol.cmima.csic.es/castresana/Gblocks_server.html

• Write a script *pir2fasta.scr* that converts a *pir* formatted alignment to *fasta* formatted alignment

```
more C22.1.pir | sed -e "s/^>P1;/>/g" -e "/^$/d" -e "/^\*/d" > C22.1.fa
```

• Write a script *multalign2oneline.pl* that convert a fasta multiple alignment into a one-line per sequence alignment (or a fasta database into a fasta database where each sequence is shown in one-line)

multalign2oneline.pl:

```
#!/bin/perl
# using a fasta multiple alignment write each sequence onto one line sequence
# or a fasta database of sequences write each sequence onto one line sequence
#use cat inputfile | multalign2oneline.pl > outputfile
$i=0;
while(<>)
{   if ( m/>/ && $i > 0 ) { print "\n"; }
    if ( m/>/ ) { print $_;}
    if ( !m/>/ ) { chop; print $_; }
    $i++;
}
```
