# Supplementary material for: Designing and running an advanced Bioinformatics and genome analyses course in Tunisia
Source: PLoS Comput Biol. 2019 Jan 28;15(1):e1006373. doi: 10.1371/journal.pcbi.1006373 (PMC6349305; doi:10.1371/journal.pcbi.1006373)
Supplement: S9 Text — (PDF) [file pcbi.1006373.s009.pdf]

S9 Text: Practical sessions for Phylogeny analyses (pdf).

Bioinformatics and Genome Analyses
September 18 – December 15, 2017. Institut Pasteur Tunis
https://webext.pasteur.fr/tekaia/BCGAIPT2017.html

Consider SPO11.1.pep and SPO11.1.dna files including yeast and one fungi orthologs to construct phylogenetic trees.
We will consider the following tree construction methods in the Phylip package:
1- Neighbor joining method,
2- Parsimony method.
3- ML (Maximum likelihood) method

In all *Phylip* programs the input file is "infile" and the output file is "outfile";
(in many circumstances the *outfile* of one program is the *infile* for the next program!)

Simple procedure:
0) mutiple alignment output : SPO11.1.phy
1) copy SPO11.1.phy into *infile*
2) *Protdist* (for dna: *dnadist*)
3) copy *outfile* into *infile*
4) *neighbor*
5) read the output file *outfile*

Procedure with bootstrapping:
1) align the sequences using the "clustalw" program;
2) use "seqboot" to construct a sample of 100 multiple alignments
3) use "protdist" to construct protein distances table;
4) "neighbor" program (using NJ method)
5) Copy "outtree" in "intree"
6) use "consense" program to construct consensus tree;

1) seqboot (100 samples, seed=113);
2) copy *outfile* into *infile*
3) Protdist (proteins) - dnadist (dna seq)
4) copy *outfile* into *infile*
5) neighbor
6) copy *outtree* into *intree*
7) consens
the obtained tree is the consensus tree

Parsimony method:
Run 1
Copy SPO11.1.pep.phy into infile
protpars
Run 2
seqboot

protpars
consense

Run 3
Copy SPO11.1.dna.phy into infile
dnapars
Run 4
seqboot
dnapars
consense

ML method:
SPO11.1.pep.phy
proml

SPO11.1.dna.phy
dnaml

Fredj Tekaia (tekaia@pasteur.fr)
