# Supplementary material for: Designing and running an advanced Bioinformatics and genome analyses course in Tunisia
Source: PLoS Comput Biol. 2019 Jan 28;15(1):e1006373. doi: 10.1371/journal.pcbi.1006373 (PMC6349305; doi:10.1371/journal.pcbi.1006373)
Supplement: S10 Text — (PDF) [file pcbi.1006373.s010.pdf]

S10 Text: Practical sessions for Molecular evolutionary analyses (pdf).

Bioinformatics and Genome Analyses
September 18 – December 15, 2017. Institut Pasteur Tunis
https://webext.pasteur.fr/tekaia/BCGAIPT2017.html

• For these sessions please review the PAML user guide, before starting the following hands-on.

• Write a script *pir2fasta.scr* that converts a *pir* formatted alignment into *fasta* formatted alignment
more SPO12.252.pir | sed -e "s/^>P1;/>/g" -e "/^$/d" -e "/^*/d" > SPO12.252.fa

• Given a set of protein sequences and their corresponding nucleotide sequences (included in the same directory), write a Perl script *alignaa2dna.pl* to align dna sequences according to their corresponding amino-acids alignments as obtained by clustalw. The output alignment will be used as input to the PAML *codeml* or *yn00* program.

Note: For PAML, aligned sequences should be in *phylip text format* with at least two spaces separating the identification of the sequence and the sequence itself. The *Phylip text format* is a multiple alignment where each sequence is a fasta formatted one-line sequence. The first line of the file includes the number of sequences tab separated from the total number of position in the multiple alignment. We will denote these files with ".paml" extension (see example below: SPO12.252.fa.paml).

• We consider a cluster SPO12.252.prt of orthologous mycobacterial proteins as obtained from the comparison of 13 mycobacterial species (in this cluster the *M. leprae* gene is missing) and its corresponding gene cluster is SPO12.252.dna.

Question for the practical sessions: Are there genes in this cluster that are submitted to positive selection?
To answer this question we use the PAML codeml program that calculates the rate of non-synonymous over synonymous substitution ($\omega = d_N/d_S$) for each pair of genes in the cluster. Genes that are submitted to positive selection are those with $\omega > 1$.
For this purpose, we need to align the gene sequences at the codon level, convert this alignment into the PAML format and then apply the codeml program (see PAML user guide).

We proceed as follows:
a) align the protein sequences using clustalw:
clust.scr SPO12.252.prt & (see scripts in S8 Text)
clust.scr includes a simple command line:
clustalw -infile=SPO12.252.prt -output=PIR -align 2>&1 > /dev/null
the output file is : SPO12.252.pir

b) obtained multiple alignment is converted into a fasta formatted alignment using the *pir2fasta.scr* script or the simple command line:
sed -e "s/>P1;/>/g" SPO12.252.pir | sed -e "/^*/d" | sed -e "/^\$/d" > SPO12.252.fa1

c) convert this alignment into one-line multiple alignment:
cat SPO12.252.fa1 | multalign2oneline.pl > SPO12.252.fa

d) convert the gene cluster SPO22.252.dna into one-line sequences:
cat SPO12.252.dna | multalign2oneline.pl > SPO12.252.fn

e) splitSPO12.252.fn into individual sequences using the *splitfasta.pl* script (seeBCGAIPT2017_msaPS.docx)
splitfasta.pl SPO12.252.fn &
f) use the *alignaa2dna.pl* script to align the dna sequences at the codon level and write the final alignment in paml format.
alignaa2dna.pl SPO12.252.fa &
the output file is SPO12.252.fa.paml

The last step is to apply *codeml* or *yn00* from the *PAML* package.
Application of these programs needs a control file (see PAML documentation and the default control file *codeml.ctl*).
g) codeml control file
Important parameters in this control file include:
seqtype = 1 : corresponding to codons and pair-wise comparison
codonFreq = 2 : corresponding to the frequencies F3x4
model = 0 : assumes one d_N/d_S ratio, pairwise comparison and codon frequencies: F3x4

the data correspond to codons and one ratio d_N/d_S per pair of sequences.

Two parameters corresponding to the input and output files, should be adapted:
seqfile = SPO12.252.fa.paml
outfile = SPO12.252.fa.paml.codeml

A shell script *sedcodeml.ctl* takes the default control file and substitutes *seqfile* and *outfile* with the correct files.
sedcodeml.ctl SPO12.252.fa.paml &

```
#!/bin/sh
cp  /home0/gensoft/PAML/paml/codeml.ctl  X.ctl
sed -e "s/seqfile.*/   seqfile = $1/g" -e "s/outfile.*/outfile = $1.codeml/g" X.ctl > $1.ctl
rm X.ctl
```

h) run codeml
codeml SPO12.252.fa.ctl &

See output results in PAML_Exp in the directory DATA:
SPO12.252.fa.paml.codeml and

i) convert these results into a table form showing the following:
Fam   seq1   seq2   ns   ls   t   S   N d_N/d_S d_N d_S
(see script: *cleancodeml.pl*)

(see PAML user guide: Fam=cluster identification(SPO12.252, seq1, seq2: pair of gene identifications in the cluster; ns: total number of genes; ls: total common alignment sequence length; t: time (distance), measured the expected number of nucleotide substitution per codon; S, N: number of synonymous substitution per synonymous site respectively number of non-synonymous sites per non-synonymous site; $\omega = d_N/d_S$: ratio of the number of non-synonymous substitution over the number of synonymous substitutions; d_S, d_N: number of synonymous respectively non-synonymous substitutions).

Final table results is shown in: SPO12.252.fa.paml.CML (in PAML_Exp directory).

Note that no pair of genes are submitted to positive selection since all pairs show $d_N/d_S < 1$.

Fredj Tekaia (tekaia@pasteur.fr)
