# Supplementary material for: Designing and running an advanced Bioinformatics and genome analyses course in Tunisia
Source: PLoS Comput Biol. 2019 Jan 28;15(1):e1006373. doi: 10.1371/journal.pcbi.1006373 (PMC6349305; doi:10.1371/journal.pcbi.1006373)
Supplement: S11 Text — (PDF) [file pcbi.1006373.s011.pdf]

S11 Text: Practical sessions on examples of characterization of some complete genomes (pdf).

Bioinformatics and Genome Analyses
September 18 – December 15, 2017. Institut Pasteur Tunis
https://webext.pasteur.fr/tekaia/BCGAIPT2017.html

Consider the *Saccharomyces* cerevisiae genome FASTA formatted databases: GSACE.seq, GSACE.dna , GSACE.pep, corresponding respectively to the complete genome sequence, the complete set of coding and protein sequences.

Write a script (*freqnt1line.pl*) to compute:
- genome base composition; genome GC% and genome size (bases);

-*freqnt1linebyseq.pl* : base composition, GC% per sequence and sequence size (total bases) in GSACE.dna;

-*freqaa1linebyseq.pl* to compute aa compositions of protein sequences in a fasta database (exp. GSACE.pep).

-*freqaa1line.pl* to compute aa compositions of a proteome (exp. GSACE.pep)

-Write a script (*extractseqbyident.pl*) to extract a sequence by its identification from a fasta formatted database of sequences;
(example: extract the sequence corresponding to YAL068c from GSACE.dna)

-Write a Perl script (*countseqperchr.pl*) to calculate for each chromosome in GSACE.pep the corresponding number of sequences? Show the results in a table form.

-Insert the species code (SACE) just after the ">" in the GSACE.pep file
Sed -e "s/>/>SACE_/g" GSACE.pep > temp
Mv temp GSACE.pep

Note this is useful for recognizing the species the sequence belongs to when comparing many genomes species.

-Write a script (*splitfasta.pl*) to split all individual sequences from a fasta formatted sequence database (all protein sequences in GSACE.pep; all dna sequences in GSACE.dna)
splitfasta.pl: output sequences should be redirected to ~/home0/data/*allsaceprt.fasta* respectively ~/home0/data/*allsacedna.fasta*.
output file sequences should be of the form: seq_ident.prt for protein sequences and seq_ident.dna for dna sequences (Exp. YAL068c.prt and YAL068c.dna).

Fredj Tekaia (tekaia@pasteur.fr)
