# Supplementary material for: Designing and running an advanced Bioinformatics and genome analyses course in Tunisia
Source: PLoS Comput Biol. 2019 Jan 28;15(1):e1006373. doi: 10.1371/journal.pcbi.1006373 (PMC6349305; doi:10.1371/journal.pcbi.1006373)
Supplement: S12 Text — (PDF) [file pcbi.1006373.s012.pdf]

S12 Text: Practical sessions for Large-scale genome comparisons (pdf).

Bioinformatics and Genome Analyses

September 18 – December 15, 2017. Institut Pasteur Tunis
https://webext.pasteur.fr/tekaia/BCGAIPT2017.html

Aim of the sessions:
Compare genomes – Analyze results – intra-species Duplication – mcl clustering – inter-species Conservation. Reciprocal Best Hits (proteins from distinct species) determination – mcl clustering.
Conservation Profiles.

Important note: we will manipulate numerous and diverse large datasets, so we have to pay attention to their organization and structural hierarchical directories so that to easily navigate between them.

For these sessions, we chose to work on real data corresponding to 3 yeast *genomes:*

Species	Code	Number of protein sequences
Saccharomyces cerevisiae	SACE	5863
Candida glabrata	*CAGL*	5202
Zygosaccharomyces rouxii	ZYRO	4991

For each proteome, we will perform the following:

Intra-species comparisons:
-Compare each proteome to itself, using blastp (with adequate options);
-Get for each protein its best significant match (presented in a table form);
-Get for each protein all its significant matches (presented in a table form);
-For each protein, calculate the number of its significant matches (presented in a table form);
-mcl clustering into families of non-unique proteins.

Interspecies comparisons:
-Perform all pair-wise proteome comparisons;

For each pair:
-Get for each protein its best significant hit in each of the cognate proteomes;
-Get for each protein all its significant hits in each of the cognate proteomes;
-For each protein calculate the number of its significant matches (presented in a table form);

Multiple comparisons:
-Extract all pairs of proteins that are Reciprocal Best Hits;
-Construct clusters (families) of orthologs using the RBH pairs and mcl;
-Construct a conservation Profile (phylogenetic profile) of each protein;
-Construct a corresponding numerical conservation profile;

Plan:

- We will use 3 yeast species: SACE, CAGL, ZYRO and corresponding complete genome sequence, predicted coding genes and proteins. We will mainly use protein sequences.

We will perform the following:

-Insert the species code before each sequence identification in proteomes and coding sequence databases. This is useful for recognizing the species corresponding to each sequence.

- Split each fasta formatted sequence database into individual sequences

- Format a database for use with *blastp*

- Run *blastp* for intra and inter species proteome comparisons

- Automate *blastp* similarity search
Use individual protein sequences of a given GDB.pep to compare each sequence to a whole sequence database (intra-inter comparisons).

- Extraction of the list of matches (blast)
- best matches for a given sequence
- all matches for a given sequence;
- list of single (orphan) proteins in each proteome

The global view of the final working directories structure is as follows:

~/home0/data includes the considered databases and individual sequences (see below)
exp. GSACE.pep and allsaceprt.fasta directory

~/home0/genanal/genomes (#working directory for genomes analyses)

SACE	CAGL	ZYRO	RBH
SACEseqnew/MCL	CAGLseqnew/MCL	ZYROseqnew/MCL	sacecagl.rbh
SACEresblp	CAGLresblp	ZYROresblp	sacezyro.rbh
CAGLseqnew	SACEseqnew	SACEseqnew	zyrocagl.rbh
CAGLresblp	SACEresblp	SACEresblp	MCL
ZYROseqnew	ZYROseqnew	CAGLseqnew	
ZYROresblp	ZYROresblp	CAGLresblp	

Sub-directories in each of these directories will be created.

Databases:

• Note the directory and corresponding pathways to the protein databases.
Databases and corresponding protein sequences should be in the ~/home0/*data* directory.

$PATH should be updated in the ".bashrc" file so that to include the path to the directory where the Blast programs are installed and the variable $BLASTDB should correspond to the directory including the Blast formatted databases.

We will move the blast programs to the directory: ~/home0/gensoft/blast/ and the blast formatted databases to the ~/home0/data/ directory.
The following two line should be added to ".bashrc" file:
export $BLASTDB=~/home0/data/
$PATH=$PATH: ~/home0/gensoft/blast/

We are considering 3 yeast genome databases:

• *Sacchromyces cerevisiae* we code SACE:
GSACE.pep, GSACE.dna, GSACE.seq.

• *Candida Glabrata* we code CAGL:
GCAGL.pep, GCAGL.dna, GCAGL.seq.

• *Zygosaccharomyces rouxii* we code ZYRO:
GZYRO.pep, GZYRO.dna, GZYRO.seq.

• Coding of the directory where to store individual protein sequences of a given species (exp of sace) is: allsaceprt.fasta
~/home0/data/allsaceprt.fasta

Comparison results:

$DIRCOMP= ~/home0/genanal/genomes/
Includes a directory per species and corresponding comparisons as subdirectories (see final organization above).

Exercises:

- fasta formatted sequences;
•) Count the number of sequences included in a fasta formatted database (GSACE.pep);

grep ">" GSACE.pep | wc -l

•) replace the identification of each sequence by adding the «genome-code_ » just after « > »
This will make easy recognition of sequence related species in the output comparisons when considering several genomes.

sed –e "s/>/>SACE_/g" /path/GSACE.pep > temp

Make sur the substitutions are correct in *temp* then: *mv temp $BLASTDB/GSACE.pep*

•) Extract from GSACE.pep, the list of sequence identifications; output file should be SACE.ident in SACEseqnew directory;

grep ">" GSACE.pep | sed –e "s/>//g" -e "s/ .//g" > SACE.ident*

I. Split the fasta formatted database into single fasta formatted sequences and formatting for blast use:

1) splitting the fasta files into individual fasta sequences
using the scripts (*splitfasta.pl* see BCGAIPT2017_CompleteGenomesPS.pdf)

• Split the GSACE.pep into single protein sequences
Redirect the output sequences (SeqIdent.prt) into the directory: *allsaceprt.fasta*

• in *~/home0/data* directory, create the directory where to store individual sequence data:
allsaceprt.fasta (sace : species code);

mkdir allsaceprt.fasta

cd allsaceprt.fasta
splitfasta.pl ../GSACE.pep (output: individual file sequences with extension ".prt": "Seqident.prt")

2) format the database for use with the BLAST programs:
makeblastdb -title «db title» –in GSACE.pep –dbtype prot

3) Perform similar splitting and reformatting for CAGL and ZYRO databases.
Expected outputs:
GCAGL.pep, allcaglprt.fasta, CAGL.ident   in the directory: $BLASTDB/CAGL/
GZYRO.pep, allzyroprt.fasta,   ZYRO.ident   in the directory: $BLASTDB/ZYRO/

II. Genome comparisons using blastp

Comparison results will be organised into (see schema above):
$DIRCOMP=~/home0/genanal/genomes/

under "~/home0/" create the directory "genanal", *cd genanal* then the directory "genome"

mkdir genanal
cd genanal
mkdir genomes

Pair-wise comparison results should be organised so that to make easy automatic navigation (scripting) between all results.
We chose the following organisation that is detailed for the comparison of SACE proteins sequences versus each of the three considered proteomes:

Results of the comparison of SACE protein sequences to the GSACE.pep database will be directed to the sub-directory: SACEseqnew:
~/home0/genanal/genomes/SACE/SACEseqnew
Whole output results of blast comparisons will be redirected to the sub-directory: SACEresblp

Similarly, comparison results of SACE protein sequences to the GCAGL.pep database will be directed to the CAGLseqnew sub-directory:
~/home0/genanal/genomes/SACE/CAGLseqnew
Detailed blastp outputs are redirected into: CAGLresblp

Finally, comparison results of SACE protein sequences versus the GZYRO.pep database will be directed to the ZYROseqnew sub-directory:
SACE/ZYROseqnew
Detailed blastp outputs go into: ZYROresblp

Similar construction should be performed for all other comparisons.

1) Reminder: using blastp, compare one protein sequence to a database

We first make sure that the blastp program and the blast formatted database are on the $PATH. Start by running the comparison of a single sequence YAL068c.prt versus the GSACE.pep database.

We assume that the *data* directory is declared:
Export BLASTDB=~/home0/data/

Standard output form:

blastp -query YAL067c.prt -db GSACE.pep -use_sw_tback –evalue 1.e-9 –seg yes –num_descriptions 300 –num_alignments 300 -out YAL067c.blp &
(note the & symbol. output file: YAL067c.blp)

blastp will use Smith-Waterman trace back option (see blastp help option), and outputs solely hits with *e-value* lower than *1.e-9* , use the *seg* filter and lists up to 300 descriptive lines.

Tabular output format:
blastp -query YAL067c.prt -db GSACE.pep -use_sw_tback –evalue 1.e-9 –seg yes –outfmt 6 -out YAL067c.blp6 &

similar to the previous run but the output is in a tabular form.

Note: The order of columns for blastp tabular output results form is: *query id, database sequence (subject) id, percent identity, alignment length, number of mismatches, number of gap openings, query start, query end, subject start, subject end, Expect value, HSP bit score.*

A- Intra-species comparison

~/home0/genanal/SACE/SACEseqnew/ (when comparing SACE with itself)
Detailed blastp results redirected into: SACEresblp

• Write a script (*blp.pl*) to compare each protein in *allsaceprt.fasta* to GSACE.pep database. The script includes the output with the "-evalue 1.e-9 -outfmt 6" options and without this option (detailed results).

-Redirect the tabular output file *allsacesace* (with the –evalue 1.e-9 –outfmt 6 options) to SACEseqnew directory, and detailed results into SACEresblp directory.

output file allsacesace stands for the comparison of all *sace* proteins versus the *sace* protein database.

Run the script:
nohup blp.pl &

• Consider *allsacesace* file and write a script (*printallhits.pl*) to extract:
the subset of significant hits: all*sacesace.HS*
Note self-significant hits should not be considered (HS stands for Highly Significant).

printallhits.pl   allsacesace &
(output should be: allsacesace.HS)

-Check for HS reciprocity
Protein sequences should be reciprocally similar with significant e-values. In case of non-reciprocity, the hit should be removed and considered non-significant.
Write a script *reciprocHS.pl* to check for reciprocity:

reciprocHS.pl allsacesace.HS > temp &
check if *allsacesace.HS* and *temp* are different
diff allsacesace.HS temp
mv temp allsacesace.HS if there is any differences.

-write a script to extract all best hits (output *bestsacesace.HS*) for each query sequence;
printbesthits.pl allsacesace.HS > bestsacesace.HS &

ln –s SACE/SACEseqnew/SACE.ident

-Single protein sequences are the complement of proteins with best hits.
-single proteins (with no significant hit; output best*sacesace.NS*);
Note: NS stands for Non-Significant.

ln –s $DIRCOMP/SACE/SACEseqnew/SACE.ident
printnshits.pl bestsacesace.HS &
output: bestsacesace.NS

• Check if the total number of proteins in “bestsacesce.NS + bestsacesace.HS” is equal to the total number of proteins in SACE.ident.

• Multiple matches (hits) from allsacesace.HS :
-Number of occurrences of multiple matches per protein sequence.

Using the "allsacesace.HS" file, write a script *countmmhits.pl* to compute the frequency of multiple matches per protein sequence.
The output file should be a table of the form:
sequence identification "tabulation" number of occurrences of multiple hits.

countmmhits.pl allsacesace.HS > freqorfsace.sace &
output is in *freqorfsace.sace.*

-sort this table in decreasing order of counts:
sort –n –k 2 -r freqorfsace.sace > freqorfsortedsace.sace &

-construct a histogram for each occurrence of multiple matches
exp. How many orfs have only one match, how many have 2 matches and so on ..

• Compute the duplication rate in SACE genome.
d_rate = (#duplicated SACE genes/total_number_of_genes in SACE)

B- Inter-species comparisons:

Compare SACE to CAGL and to ZYRO.

• under the directory SACE create the directories CAGLseqnew and CAGLresblp

mkdir CAGLseqnew
mkdir CAGLresblp

• use *blp.pl* script to compare all SACE proteins to GCAGL.pep:

-redirect the tabular output (with the –evalue 1.e-9 –outfmt 6 options) to allsacecagl file into the CAGLseqnew directory.

• *mv allsacecagl allsacecagl.HS*

• from *allsacecagl.HS* file extract subsets corresponding to:
-all best significant hits (output *bestsacecagl.HS*) i.e. conserved proteins.

printbesthit.pl allsacecagl.HS &
(output is in bestsacecagl.HS)

• non conserved proteins (with no significant hit; output *bestsacecagl.NS*);

ln –s $DIRCOMP/SACE.ident
printnshits.pl SACE.ident bestsacecagl.HS &
(output in *bestsacecagl.NS)*

-using *allsacecagl.HS,* construct a table of multiple matches per sequence:
protein (tab) occurrences_of_possible_matches;
countmmhits.pl allsacecagl.HS > freqorfsace.cagl &

-Construct a table showing frequencies of multiple matches:
Query protein (tab) occurrence of multiple matches
sort –n –k 2 –r freqorfsace.cagl > freqorfsortedsace.cagl &

• Calculate the conservation rate of SACE genome into CAGL genome.
c_rate = (#conserved SACE genes in CAGL)/(total number of genes in SACE)

• Perform similar comparisons and computations with ZYRO
• Perform the same comparisons and computations for CAGL and ZYRO genomes (intra-species and inter-species comparisons) starting in paragraph II.

III. Multiple comparisons

-Extract all pairs of proteins that are Reciprocal Best Hits to be used for mcl clustering;

Write a script (rbh.pl) that extracts pairs of proteins from distinct species and that are reciprocal best hits

• Change to directory RBH

make links to bestxxyy.HS files
ln –s ../SACE/CAGLseqnew/bestsacecagl.HS
ln –s ../CAGL/SACEseqnew/bestcaglsace.HS

rbh.pl bestsacecagl.HS bestcaglsace.HS &
output file *is sacecagl.rbh*

ln –s ../SACE/ZYROseqnew/bestsacezyro.HS
ln –s ../ZYRO/SACEseqnew/bestzyrosace.HS

rbh.pl bestsacezyro.HS bestzyrosace.HS &
output file is *sacezyro.rbh &*

ln –s ../ZYRO/CAGLseqnew/bestzyrocagl.HS
ln –s ../CAGL/ZYROseqnew/bestcaglzyro.HS

rbh.pl bestzyrocagl.HS bestcaglzyro.HS &
output file is *zyrocagl.rbh&*

• merge the 3 rbh files into *alltotorth* and add sequence size

• extract columns 1 and 3 into one column (temp)

• *countmmhits.pl temp freqtotorth &*

• frequency table of orthologous genes sorted in decreasing occurrences;
(*sort –n –k 2 –r temp > freqtotorth &)*

Practical sessions continue with (see S13 Text).

Fredj Tekaia (tekaia@pasteur.fr)
