# Supplementary material for: Designing and running an advanced Bioinformatics and genome analyses course in Tunisia
Source: PLoS Comput Biol. 2019 Jan 28;15(1):e1006373. doi: 10.1371/journal.pcbi.1006373 (PMC6349305; doi:10.1371/journal.pcbi.1006373)
Supplement: S13 Text — (PDF) [file pcbi.1006373.s013.pdf]

S13 Text: Practical sessions for Large-scale genome comparisons and Paralogs, Orthologs inference and clustering (pdf).

Bioinformatics and Genome Analyses
September 18 – December 15, 2017. Institut Pasteur Tunis
https://webext.pasteur.fr/tekaia/BCGAIPT2017.html

See first: S12 Text

mcl web page :
http://micans.org/mcl/

Paralogs - Orthologs
-*mcl* clustering of non-unique proteins (paralogs)

-list of Reciprocal Best Hits (*rbh*) as deduced from inter-species proteome comparisons

-*mcl* clustering of orthologs as obtained from *rbh* pairs

-*meme* analyses (motifs search) from clusters of paralogs and orthologs

The global view of the final working directories structure is as follows:

~/home0/genanal/genomes (#working directory for genomes analyses)

SACE	CAGL	ZYRO	RBH
SACEseqnew/MCL	CAGLseqnew/MCL	ZYROseqnew/MCL	sacecagl.rbh
SACEresblp	CAGLresblp	ZYROresblp	sacezyro.rbh
CAGLseqnew	SACEseqnew	SACEseqnew	zyrocagl.rbh
CAGLresblp	SACEresblp	SACEresblp	MCL/mclorthfamilies
ZYROseqnew	ZYROseqnew	CAGLseqnew	MCL/FAMSEQ
ZYROresblp	ZYROresblp	CAGLresblp	

A- Intra-species comparison

• Classification of non-unique genes in SACE into families of paralogs using *mcl*

• make sure the *mcl* program is on your $PATH
We need the *SACE.ident* and the *allsacesace.HS* files

mcl needs (see *mcl* manual) a numerical matrix that includes a score in each cell corresponding to the similarity between the corresponding orfs pair (line, column).

Available scripts for the preparation of this matrix and for running *mcl* as well as the extraction of the obtained clusters, should be run as shown below.

During the practical session we will discuss each of the steps involved in this procedure.

• in SACEseqnew create a MCL directory

• scripts that will be used with *mcl* need a specific format for the file *allsacesace.HS*.

For this reason we create a new file called *allsaceseqnew.HS* that includes a subset of columns: Query_orf, Size, Hit_orf, “HS” and e-value

0) *extractallHSval.pl allsacesace.HS &*
The outfile is *allsaceseqnew.HS*

cd MCL;
make a symbolic link to *allsaceseqnew.HS* file
ln –s ../allsaceseqnew.HS

From the calculated *freqorfsace.sace* (see BCGAIPT2017_GenomCompPS.pdf) file :
1) *sort -n -k 2 -r ../freqorfsace.sace | nom.pl > nomorf*
(*nom.pl* is a script that extracts the first column from a file i.e. the sequence identifications column in this case). The output file *nomorf* includes ORF identifications in corresponding multiple matches decreasing order.

Note: The following prewritten scripts are available for use by the students:
Associate an index number to each ORF identification:
2) *mcltabform.pl nomorf > SACE.tab*

Replace the ORF identification by its corresponding index (in *SACE.tab*) and add a column including the *log(e-value)* corresponding to the log values of the e-value.
3) *mclall2num.pl SACE.tab allsaceseqnew.HS > allsacenum*

Transform the allsacenum to the *mcl cmi* format
(see also: Enright AJ, Van Dongen S. and Ouzounis C. (2002. An efficient algorithm for large-scale of protein families. Nucleic Acids Res. 30(7):1575-84.)
4) *mclall2cmi.pl allsacenum SACE.tab & (output allsacenum.cmi)*

Apply the *mcl* program with inflation index (-I 3.0) and the output file SACE.clusters:
5) *mcl allsacenum.cmi -i 3.0 -o SACE.clusters & (mcl clustering)*

mcltribefamilies.pl is written by Enright AJ (see above indicated reference) to construct tribe-clusters from the mcl clusters.
6) *mcltribefamilies.pl SACE.clusters SACE.tab > SACE.clusters-tribe &*

For each ORF print its corresponding cluster and size in the following order:
Cn.m <tab> n <tab) ORF identification, where C (stands for Cluster), n is the number of elements in the cluster and m is in arbitrary index order to differentiate clusters with identical size. The last column is the ORF identification.

Note that m values for each size are arbitrarily indicated to distinguish between cluster of identical sizes.
7) *mclclustsize.pl SACE.clusters-tribe > SACE.mclclusters &*

Renumber classes in increasing order for each size.
Put m values in increasing order (starting from 1) for each size.
8) *renumclass.pl SACE.mclclusters &*

Histogram of cluster contents: compute how many clusters are constructed for each cluster size compute.

Extract the first column (using *nom.pl*) and keep unique identifications, then keep solely the size after "C" by remove the "C" and all characters after the dot. The output file *temp* includes the sorted list of sizes.
1) *more SACE.mclclusters | nom.pl | sort –u | sed –e "s/\..*//g" –e "s/C//g" | sort –n > temp*

The script *freqsortednames.pl* calculates the frequency per size:
2) *freqsortednames.pl temp&* (output file *temp.freq*)

Add "C" at the first position of the distinct cluster sizes:
3) sed –e "s/^/C/g" temp.freq > SACE-mclclusters.histo

9) Extract from SACE.mclclusters each cluster and its corresponding members.

Clusters should be of the form: *Cn.m.mcl* where n is the size (number of elements) of the cluster and m is an arbitrary order. The first line includes Cn. Each of next line includes: orf, cluster_size and mcl_cluster (tab separated).
Individual cluster files should be redirected to a directory *mclfamilies*.

In MCL create a directory mclfamilies
mkdir mclfamilies;
cd mclfamilies

../extractmclcluster.pl ../SACE.mclclusters&

10) From the file SACE.mclclusters and SACE.ident, create a file *SACE.mclpar* for cluster of paralogs including for each orf:
orf_ident tab mclcluster (Cp.q or single)
mclcluster is the identification of the cluster including the orf_ident or "single" if orf_ident is unique in its genome

-Perform similar computations in CAGL and ZYRO.
So that to obtain mcl clusters of non-unique proteins in CAGL (CAGL.mclclusters) and in ZYRO (ZYRO.mclclusters), their corresponding families as well as CAGL.mclpar and ZYRO.mclpar.

III. Multiple comparisons

-Extract all pairs of proteins that are Reciprocal Best Hits and mcl clustering;

-mcl clustering of orthologous genes (RBH orfs are assumed orthologs)

Under the directory genomes create a directory RBH.
mkdir RBH
From the file alltotorth (see BCGAIPT2017_GenomComPS.pdf) construct a table *alltotorth.HS* similar to *allsaceseqnew.HS*
then apply the mcl procedure used for intra-species clustering of paralogs.

For this, we create a new file called *alltotorth* that includes a subset of columns: Query_orf, Size, Hit_orf, HS, e-value (Query_orf and Hit_orf are rbh orfs and are assumed orthologs)

0) *tt.pl alltotorth > temp*; mv temp *alltotorth*

Create a directory mcl:
mkdir MCL
cd MCL;
ln –s ../alltotorth

cat alltotorth | nom.pl | sort > temp

freqsortednames.pl temp & (output file is *temp.freq*).
mv temp.freq freqtotorth.freq

1) *sort -n -k 2 -r ../freqtotorth.freq | nom.pl > nomorf*
(*nom.pl* is a script that extracts the first column from a table i.e. the sequence identifications column in this case. Nomorf are in decreasing order of occurrences of multiple matches).

Use the same procedure as for the clustering of paralogous ORFs, with ORTH replacing the species identification:
2) *mcltabform.pl nomorf > ORTH.tab*

3) *mclall2num.pl ORTH.tab alltotorth > allorthnum*

4) *mclall2cmi.pl allorthnum ORTH.tab & (output allorthnum.cmi)*

5) *mcl allorthnum.cmi -I 3.0 -o ORTH.clusters & (mcl clustering)*

6) *mcltribefamilies.pl ORTH.clusters ORTH.tab > ORTH.clusters-tribe &*

7) mclclustsize.pl ORTH.clusters-tribe > ORTH.mclclusters &

Renumber classes in increasing order for each size:
8) *renumclass.pl ORTH.mclclusters &*

9) extract from ORTH.mclclusters each cluster with its corresponding members.
Clusters should be of the form: Cn.m.mcl where n is the number of elements in the cluster and m is an arbitrary order. The first line includes Cn. Each next line includes: orf, cluster_size and mcl_cluster (tab separated).
Use the script: *extractmclcluster.pl*

In MCL create a directory mclorthfamilies
mkdir mclorthfamilies;
cd mclorthfamilies

10) ../extractmclcluster.pl ../ORTH.mclclusters&

-Join paralogs and orthologs clusterings for each orf

For each orf (in *nomorf*) in the orthologs cluster add its corresponding paralogs cluster or single (as obtained in SACE.mclpar, CAGL.mclpar and ZYRO.mclpar).
Output file should be of the form: orf_ident (tab) Cn.m-Cp.q (Cn-m orthologs cluster and Cp.q paralogs cluster or "single") (*allorthpar.pl*).
Output file: sfamORTH-MCL

-Protein sequences for each cluster of ortholgs

-Under the directory MCL create a directory FAMSEQ
mkdir FAMSEQ

For each cluster in *mclorthfamilies* insert corresponding protein sequences into a *Cn.m-Cp.q.pep* file
catclust.pl sfamORTH-MCL& output should be in FAMSEQ.

-meme/mast analysis of orthologous clusters

For a given cluster of protein sequences search for motifs using *meme*.
0) meme $file -protein -oc . -nostatus -time 18000 -maxsize 60000 -mod zoops -nmotifs 15 -minw 50 -maxw 50

1) mast meme.xml $file –oc . -nostatus

-Construct a conservation profile (or phylogenetic profile) for each protein;
For each protein sequence of the three species construct a conservation profile

	SACE	CAGL	ZYRO
consprof	x	y	z
……………………………………….			
consprof01	1	0	1
consprof02	0	1	1
consprof03	1	1	1
……………………………………….			

We consider the species following order : SACE, CAGL and ZYRO.

CP(i,j)=1 if i $\in$ j or i is conserved in j, i is an orf product identifier and j is a species
CP(i,j)=0 otherwise.

consprofile.pl SACE CAGL ZYRO
consprofile.pl CAGL SACE ZYRO
consprofile.pl ZYRO SACE CAGL

ln –s $DIRCOMP/SACE/SACE.ident
consprofile.pl SACE.ident sacesace.rbl sacecagl.rbh sacezyro.rbh &
outfiles: SACE_ConsProf and SACE_ConsProf01

ln –s $DIRCOMP/CAGL/CAGL.ident
consprofile.pl CAGL.ident caglcagl.rbh caglsace.rbh caglzyro.rbh &
outfiles: CAGL_ConsProf and CAGL_ConsProf01

ln –s $DIRCOMP/ZYRO/ZYRO.ident
consprofile.pl ZYRO.ident zyrozyro.rbh zyrosace.rbh zyrocagl.rbh &
outfiles ZYRO_ConsProf and ZYRO_ConsProf01

Fredj Tekaia (tekaia@pasteur.fr)
