# Supplementary material for: Designing and running an advanced Bioinformatics and genome analyses course in Tunisia
Source: PLoS Comput Biol. 2019 Jan 28;15(1):e1006373. doi: 10.1371/journal.pcbi.1006373 (PMC6349305; doi:10.1371/journal.pcbi.1006373)
Supplement: S14 Text — (PDF) [file pcbi.1006373.s014.pdf]

S14 Text: Practical sessions for Tandem repeated motifs search (pdf).

Bioinformatics and Genome Analyses
September 18 – December 15, 2017. Institut Pasteur Tunis
https://webext.pasteur.fr/tekaia/BCGAIPT2017.html

Aim of the session:

1- Connect to the server https://tandem.bu.edu/trf/trf.html and download the "Linux command line 64 bits" version into your ~/bin/ directory and rename it *trf*.

2- Make a directory TRF where to work during this session (same level as RBH).

3- Data to be considered: GSACE.dna, GCAGL.dna, and GZYRO.dna.

4- Search for repeated motifs in genes:
-consider first, 2 simple examples: YDR204w.dna, YDR545.dna
-search for tandem motifs using the *trf* server than the local *trf*.

5- Write a script to search for tandem motifs in all SACE gene sequences (included in allsacedna.fasta directory)

6- Write a script to show results into a table form.

7- Look for genes including megasatellites (Megasatellites are motifs with size >=90nt).

Reminder (G. Benson (1999) "Tandem repeats finder: a program to analyze DNA sequences". *NAR:27 pp. 573-580.*):

use: *trf file match mismatch delta pm pi minscore maxperiod*
where
file = dna sequence(s) input file (fasta)
match = matching weight
mismatch = mismatching penalty
delta = indel penalty
pm = match probability (whole number)
pi = indel probability (whole number)
minscore = minimum alignment score to report
(exp: 50 assuming perfect alignment. Need to align at least 5 characters to meet the minimum score (5 copies of 5 char motif)
maxPeriod = maximum period size to report

The recommended values for Match, Mismatch and Delta are 2, 7, and 7 respectively.
trf inputfile.fa 2 7 7 80 10 50 2000 -d -m -h
-d : output text file, -m: mask input seq and put "N" in repeated positions,
-h : no html output file

Example of command line:

trf CAGL0A01067g.fa 2 7 7 80 10 50 2000 -d -m -h
and corresponding output:

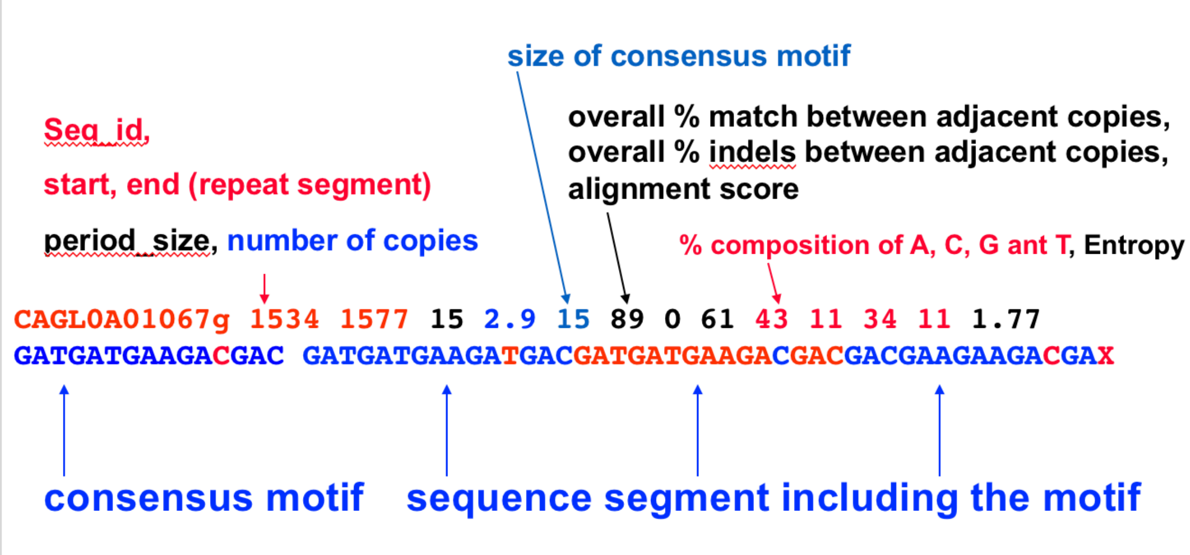

Other examples:
Seq_Id, start, end (repeat segment), period_size, number of copies, size of consensus motif, overall % match between adjacent copies, overall % indels between adjacent copies, alignment score, % composition of A, C, G ant T, Entropy, motif, segment

CAGL0A01067g 1534 1577 15 2.9 15 89 0 61 43 11 34 11 1.77 GATGATGAAGACGAC
GATGATGAAGATGACGATGATGAAGACGACGACGAAGAAGACGA

CAGL0A01193g 113 147 12 2.9 12 100 0 70 51 31 8 8 1.63 AGAACATCACAC AGAACATCACACAGAACATCACACAGAACATCACA

CAGL0A01001g 534 586 15 3.5 15 92 0 88 33 32 20 13 1.91 TCAGCAAGCTCAAGC
TCAGCAAGCTCAGGCTCAGCAAGCTCAAACTCAGCAAGCTCAAGCTCAGCAAG
CAGL0A01001g 565 651 42 2.1 42 88 0 129 37 32 18 11 1.86 CAGCAAGCTCAAGCTCAGCAAGTACCACAGTACAGAACACAA
CAGCAAGCTCAAGCTCAGCAAGTACCACAGTACCGTACACAACAGCAAGCTCCAGCTCAGCAAGTACCACAGTATAGAACGCAACAG

CAGL0A01284g 1856 2048 51 3.8 50 73 14 162 30 6 34 28 1.83
TGGATGAGAACGGTGATTGTGACTATAGTTGTAAAAAGACGGTGGTAAGT
TGGATGAGAACGGTGATTGTGACTATAGTTGTAAAAATGGCGGTGGTAAGTTGGATGAAGACGGTAATTGTGATATGTCCTGTAAAGA
GAACGGCGGTCAGTTGGATGAGGATGGTGATTGTGACTTGAGCTGTAAAGAAGATGGTGGTAAGTTAGATGGTAACGGTGATTGTGAT
TATAGTTGCAAAGAAGA
CAGL0A01284g 1856 2045 102 1.9 101 79 10 217 29 6 34 28 1.83
TGGATGAGAACGGTGATTGTGACTATAGCTGTAAAAATGACGGTGGTAAGTTAGATGAAGACGGTAATTGTGATATGTCTGCAAAGAG
AACGGCGGTCAGT
TGGATGAGAACGGTGATTGTGACTATAGTTGTAAAAATGGCGGTGGTAAGTTGGATGAAGACGGTAATTGTGATATGTCCTGTAAAGA
GAACGGCGGTCAGTTGGATGAGGATGGTGATTGTGACTTGAGCTGTAAAGAAGATGGTGGTAAGTTAGATGGTAACGGTGATTGTGAT
TATAGTTGCAAAGA

4- Search for repeated motifs in genes:
-consider first, 2 simple examples: YDR204w.dna, YDR545w.dna
-search for tandem motifs using the *trf* server than the local *trf*.

trf YDR204w.dna 2 7 7 80 10 50 2000 -d -m -h > YDR204w.trf
trf YDR545w.dna 2 7 7 80 10 50 2000 -d -m -h > YDR545w.trf

5- Write a script to search for tandem motifs in all SACE gene sequences (included in allsacedna.fasta directory). Output should directed to gene_ident.trf.

```
#!/bin/sh
For file in `ls $path/allsacedna.fasta/*.dna`
do
FILE= `echo $file | sed -e "s/\/.*fasta\///g" -e "s/\.dna//g"`
trf  $file 2 7 7 80 10 50 2000 -d -m -h  > $FILE.trf
done
```

6- concatenate results into a single file SACE.trf.

```
cat Y*.trf >> SACE.trf
```

7- Look for genes including megasatellites (Megasatellites are motifs with size >=90nt).

Following the detailed example shown with CAGL0A01067g.fa the size of the consensus tandemly repeated motif is at position 6.

```
#!/bin/perl
$IN=@ARGV[0]; #input file SACE.trf
$OUT="MEGASAT90.trf";
open (IN, "$IN");
open (OUT, ">$OUT");
while<IN>)
{
@tab=split(/\s+/,$_);
if ($tab[5] >= 90 ) {print OUT $_; }
}
close(IN); close(OUT);
```

Fredj Tekaia (tekaia@pasteur.fr)
