# Supplementary material for: Designing and running an advanced Bioinformatics and genome analyses course in Tunisia
Source: PLoS Comput Biol. 2019 Jan 28;15(1):e1006373. doi: 10.1371/journal.pcbi.1006373 (PMC6349305; doi:10.1371/journal.pcbi.1006373)
Supplement: S15 Text — (PDF) [file pcbi.1006373.s015.pdf]

S15 Text: Practical sessions for Paralogs, Orthologs inference and clustering in 5 mycobacterial proteomes (pdf).

Bioinformatics and Genome Analyses

September 18 – December 15, 2017. Institut Pasteur Tunis
https://webext.pasteur.fr/tekaia/BCGAIPT2017.html

See first: S12 Text and S13 Text.

For the bacterial genome comparison sessions we will consider 5 Mycobacterial species genomes: *Mycobacterium tuberculosis* H37R (GMYTU.seq, GMYTU.dna, GMYTU.pep), *Mycobacterium bovis* (GMYBO.seq, GMYBO.dna, GMYBO.pep), *Mycobacterium leprea* (GMYLE.seq, GMYLE.dna, GMYLE.pep), *Mycobacterium marinum* (GMYMA.seq, GMYMA.dna, GMYMA.pep) and *Mycobacterium ulcerans* (GMYUL.seq, GMYUL.dna and GMYUL.pep).

Total number of predicted proteins per species:

species	Code	Number of predicted proteins
M. tuberculosis H37R	MYTU	3996
Mycobacterium bovis AF2122/97	MYBO	3920
Mycobacterium leprae	MYLE	1614
Mycobacterium_marinum	MYMA	5483
Mycobacterium_ulcerans	MYUL	5105

Corresponding data are located in the DATA/MYCOBACT_data directory.

We will use all the scripts that have been written and used in the preparation and analyses of the yeast species. Some adaptations are needed to run adequately for the Mycobacterial species.

The global view of the final working directories structure is as follows:
~/home0/data/ (includes *.pep, *.dna, *.seq files and all*prt.fasta directories
~/home0/genanal/genomes (#working directory for genomes analyses)

MYTU	MYBO	MYLE	MYMA	MYUL	RBH
MYTUseqnew/MCL	MYBOseqnew/MCL	MYLEseqnew/MCL	MYMAseqnew	MYULseqnew	mytumybo.rbh
MYTUresblp	MYBOresblp	MYLEresblp	MYMAresblp	MYULresblp	mytumyle.rbh
MYBOseqnew	MYTUseqnew	MYTUseqnew	MYTUseqnew	MYTUseqnew	mytumyma.rbh
MYBOresblp	MYTUresblp	MYTUresblp	MYTUresblp	MYTUresblp	mytumyul.rbl
MYLEseqnew	MYLEseqnew	MYBOseqnew	MYBOseqnew	MYBOseqnew	mybomyle.rbh
MYLEresblp	MYLEresblp	MYBOresblp	MYBOresblp	MYBOresblp	mybomyma.rbh
MYMAseqnew	MYMAseqnew	MYMAseqnew	MYMAseqnew	MYMAseqnew	mybomyul.rbh
MYMAresblp	MYMAresblp	MYMAresblp	MYMAresblp	MYMAresblp	mymamyul.rbh
MYULseanew	MYULseqnew	MYULseqnew	MYULseqnew	MYULseqnew	MCL/mclorthfamilies
MYULresblp	MYULresblp	MYULresblp	MYULresblp	MYULresblp	MCL/FAMSEQ

Write script that perform automatically the comparisons and results analyses.

Data preparation

-Move the data (GMYTU.pep, GMYBO.pep,...) to the directory ~/home0/data.
-Add the species code to each protein identification

-Split these databases into individual sequences in their corresponding allxxprt.fasta directory and format each proteome for use with blast.
dataprep.pl genomelist &
where *genomelist* includes the species coding list.
-Extract orfs identifiers and redirect to $SPEC.ident

The main purpose of these practical sessions is to try to automate all the scripts that have been used for the yeast genomes to perform:
-simple description of each genome (GC% per genome and per sequence, aa compositions)
-genome comparisons using blastp
-rbh detection
-mcl clustering
-meme analyses
-conservation profiles determination.

-genome descriptions

-list of orfs identifiers in each proteome if not done above
nom.scr genomeslist &

-Base composition of a list of dna databases.
Basecomp.scr listmyco &
Where listmyco includes for example the five mycobacterial ".seq".
Note: one line per file
Output (see *basecomp.pl*) GMYTUseq.BASEcomp and GMYBO.BASEcomp including the percent base composition, the GC% and the total size.

-Amino acids compositions of many proteomes:
Write a script that uses the *freqaa1line.pl* script (see BCGAIPT2017_CompleteGenomPS.pdf) to compute the amino-acids compositions of a list of proteomes.

Freqaa1line.scr listmyco &
Where *listmyco* includes the list of the five mycobacterial proteomes ".pep".

-Intra-species and inter-species comparisons:

blastp comparisons
write a script *compareallvsall.scr* that performs all pairwise *blastp* comparisons between the 5 mycobacterial species.
Start by writing the script that performs the comparison of one species versus a list of species:
comparenewg2eachg.pl $SPEC genomeslist &
$SPEC is the species to be compared to each of the species proteomes in *genomelist*.
genomelist includes the list of codes of the considered species.
This script uses the *blastallgenomes.scr* that performs the comparisons and stores the results in the corresponding directories following the schema outlined above.

The script *blastallgenomes.scr* performs the following steps:

For each comparison the *allxxyy, allxxyy.HS and bestxxyy.HS* files are constructed in the corresponding directory.
allsaceseqnew.HS for the use of *mcl*, is also created and includes a subset of columns: Query_orf, Size, Hit_orf, "HS" and e-value

compareallvsall.scr genomeslist &
calls comparenewg2eachg.pl to perform all pair-wise comparisons.

Reciprocal Best Hits (rbh): intra and inter-species
-intra-species: extract the set of significant hits, check for reciprocity and extract the list of the best hits. Consider *printallhits.pl*, *reciprocHS.pl* and printbesthits.pl scripts
printallhits.scr genomeslist &

-inter-species: extract best hits from allxxyy
printbesthits.pl

-list of Reciprocal Best Hits (*rbh*) as deduced from inter-species proteome comparisons
multrbh.pl genomelist &
Pair-wise rbh hits are computed (xxyy.rbh) and redirected to the directory : RBH.

Clustering of Paralogs and of Orthologs
-mcl clustering of paralogs (non-unique proteins) in each species:
mclpar.pl genomeslist &
This script performs the following steps:

for a given species code $GEN:
• in $GENseqnew create an MCL directory

• scripts that will be used with *mcl* need a specific format for the file allgengen.HS.

For this reason we create a new file called *all$genseqnew.HS* that includes a subset of columns: Query_orf, Size, Hit_orf, "HS" and e-value

0) *extractallHSval.pl allgengen.HS &*
The outfile is *all$genseqnew.HS*

cd MCL;
make a symbolic link to all$genseqnew.HS file
ln –s ../all$genseqnew.HS

From the calculated *freqorf$gen.$gen* (see BCGAIPT2017_GenomCompPS.pdf) file :
1) *sort -n -k 2 -r ../freqorf$gen.$gen | nom.pl > nomorf*
(*nom.pl* is a script that extracts the first column from a file i.e. the sequence identifications column in this case). The output file *nomorf* includes ORF identifications in corresponding multiple matches decreasing order.

Note: The following prewritten scripts are available for use by the students:

Associate an index number to each ORF identification:
2) *mcltabform.pl nomorf > $GEN.tab*

Replace the ORF identification aby its corresponding index (in $GEN.tab) and add a column including the log(e-value) corresponding to the log values of the e-value.
3) *mclall2num.pl $GEN.tab all$genseqnew.HS > all$gennum*

Transform the all$gennum to the *mcl cmi* format
(see also: Enright AJ, Van Dongen S. and Ouzounis C. (2002. An efficient algorithm for large-scale of protein families. Nucleic Acids Res. 30(7):1575-84.)
4) *mclall2cmi.pl all$gennum $GEN.tab & (output all$gennum.cmi)*

Run the *mcl* program with inflation index (-i 3.0) and the output file $GEN.clusters:
5) *mcl all$gennum.cmi -i 3.0 -o $GEN.clusters & (mcl clustering)*

mcltribefamilies.pl is written by Enright AJ (see above indicated reference) to construct tribe-clusters from the mcl clusters.
6) *mcltribefamilies.pl $GEN.clusters $GEN.tab > $GEN.clusters-tribe &*

For each ORF print its corresponding cluster and size in the following order:
Cn.m <tab> n <tab) ORF identification, where C (stands for Cluster), n is the number of elements in the cluster and m is in arbitrary index order to differentiate clusters with identical size. The last column is the ORF identification.
Note that m values for each size arbitrarily indicated.
7) *mclclustsize.pl $GEN.clusters-tribe > $GEN.mclclusters &*

Renumber classes in increasing order for each size.
Put m values in increasing order (starting from 1) for each size.
8) *renumclass.pl $GEN.mclclusters &*

Histogram of cluster contents: compute how many clusters are constructed for each cluster size compute.

Extract the first column (nom.pl) and keep unique identifications, then keep solely the size after "C" by remove the "C" and all characters after the dot. The output file *temp* includes the sorted list of sizes.
1) *more $GEN.mclclusters | nom.pl | sort –u | sed –e "s/\..*//g" –e "s/C//g" | sort –n > temp*

The script *freqsortednames.pl* calculates the frequency per size:
2) freqsortednames.pl temp& (output file temp.freq)

Add "C" at the first position of the distinct cluster sizes:
3) sed –e "s/^/C/g" temp.freq > $GEN-mclclusters.histo

9) Extract from $GEN.mclclusters each cluster and its corresponding members.

Clusters should be of the form: Cn.m.mcl where n is the size (number of elements) of the cluster and m is an arbitrary order. The first line includes Cn. Each of next line includes: orf, cluster_size and mcl_cluster (tab separated).
Individual cluster files should be redirected to a directory *mclfamilies*.

In MCL create a directory mclfamilies
mkdir mclfamilies;
cd mclfamilies

../extractmclcluster.pl ../$GEN.mclclusters&

10) From the file $GEN.mclclusters and $GEN.ident, create a file *$GEN.mclpar* for cluster of paralogs including for each orf:
orf_ident <tab< mclcluster (Cp.q or single)
mclcluster is the identification of the cluster including the orf_ident or “single” if orf_ident is unique in its genome

End of the script mclpar.pl

-*mcl* clustering of orthologs as obtained from *rbh* pairs
working directory is RBH:
we assume rbh orfs are orthologs.
merge all rbh files.
cat *.rbf >> alltotorth

The script *mclorth.pl* performs the following steps:

Create a directory mcl:
mkdir MCL
cd MCL;
ln –s ../alltotorth

cat alltotorth | nom.pl | sort > temp

freqsortednames.pl temp & (output file is *temp.freq*).
mv temp.freq freqtotorth.freq

1) *sort -n -k 2 -r ../freqtotorth.freq | nom.pl > nomorf*
(nomorf includes sequence identifiers in decreasing order of occurrences of multiple matches).

Use the same procedure as for the clustering of paralogous ORFs, with ORTH replacing the species identification:
2) *mcltabform.pl nomorf > ORTH.tab*

3) *mclall2num.pl ORTH.tab alltotorth > allorthnum*

4) *mclall2cmi.pl allorthnum ORTH.tab & (output allorthnum.cmi)*

5) *mcl allorthnum.cmi -I 3.0 -o ORTH.clusters & (mcl clustering)*

6) *mcltribefamilies.pl ORTH.clusters ORTH.tab > ORTH.clusters-tribe &*

7) mclclustsize.pl ORTH.clusters-tribe > ORTH.mclclusters &

Renumber classes in increasing order for each size:
8) *renumclass.pl ORTH.mclclusters &*

9) extract from ORTH.mclclusters each cluster with its corresponding members.
Clusters should be of the form: Cn.m.mcl where n is the number of elements in the cluster and m is an arbitrary order. The first line includes Cn. Each next line includes: orf, cluster_size and mcl_cluster (tab separated).
Use the script: *extractmclcluster.pl*

In MCL create a directory mclorthfamilies
mkdir mclorthfamilies; cd mclorthfamilies

10) ../extractmclcluster.pl ../ORTH.mclclusters&

construct the file *nomorth.mclorth* including for each orf identifier in nomorth:
orf_ident <tab> orthologs_cluster (Cn.m)
11) *identmclorth.pl nomorf ORTH.mclclusters*

-Join orthologs and paralogs clusters for each orf

For each orf (of *nomorf*) in the orthologs cluster add its corresponding paralogs cluster or single (as obtained in *$GEN.mclpar* files). Output should be of the form: orf_ident (tab) *Cn.m-Cp.q* (*Cn.m* orthologs clusters or "single" and *Cp.q* paralogs clusters) (allorthpar.pl).
Output file: sfamorth-mcl (orf_ident <tab>Cn.m <tab> Cp.q/single<tab>Cn.m-Cp.q)

allorthpar.pl nomorth.mclorth &

-Search for motifs in clusters of paralogs and orthologs using meme

-create a directory *FAMSEQ*

-For each cluster in *mclorthfamilies* append corresponding orf protein sequences into a *Cn.m.pep* file: *catclust.pl sfamorth-mcl &*   output should be in FAMSEQ.

allorthpar.pl nomorth.mclorth &

-write a script *meme.scr* to search for motifs in each family in *sfamorth-mcl*.

meme.scr performs the following two steps:

0) meme $file -protein -oc . -nostatus -time 18000 -maxsize 60000 -mod zoops -nmotifs 15 -minw 50 -maxw 50
1) mast meme.xml $file –oc . -nostatus

Fredj Tekaia (tekaia@pasteur.fr)
