# Supplementary material for: Designing and running an advanced Bioinformatics and genome analyses course in Tunisia
Source: PLoS Comput Biol. 2019 Jan 28;15(1):e1006373. doi: 10.1371/journal.pcbi.1006373 (PMC6349305; doi:10.1371/journal.pcbi.1006373)
Supplement: S16 Text — This document includes the questionnaire that has been completed by each participant at the end of the course to evaluate the different steps of the course development and realization. The questionnaire ends with offering the inclusion of free comments. (DOCX) [file pcbi.1006373.s016.docx]

**S16 Text: Evaluation questionnaire**

This document includes the questionnaire that has been completed by each participant at the end of the course to evaluate the different steps of the course development and realization.

It has been completed by each participant at the end of the course.

The evaluation consisted of assigning a qualification among “Excellent”, “Very good”, “Good”, “Adequate”, “Poor” and “Unsatisfactory” to questions related to the different steps of the course development:

a) The course announcement and environment,

b) The quality of the scientific talks and practical sessions as well as the series of lectures concluding the course,

c) The Lab meetings organization and interest,

d) The organizational aspects of the course,

e) The whole course program,

f) Overall evaluation of the three months ‘course.

The questionnaire ends with offering the inclusion of free comments.

**Evaluation Questionnaire**

**Bioinformatics and Genome Analyses Course**

**September 18 – December 15, 2017**

**Institut Pasteur Tunis**

We kindly ask you to provide your feedback on the three-months course by **carefully completing** this questionnaire. Please send it latest on **Sunday December 10, 2017, 5:00 pm**.

Results will be presented on Tuesday 12,2:00 pm.

**Before starting the completion of this questionnaire, please read carefully the course announcement, the Recommendations document and the course program: https://webext.pasteur.fr/tekaia/BCGAIPT2017.html.**

Your First name:

Your Family name:

Gender:

e-mail address:

Function (position):

Background:

Institution:

**Please give your evaluation** (tick your choice by X)**:**

**Q1.** Were the course program and organization adequately announced in terms of content and effective realization:

**1:** Adequate **2:** Not adequate

**Q2:** Was your background adequate to participate in this course:

**1:** Yes **2:** No

**Q3:** Did you have the necessary time (average 3h/day) during the course period to efficiently review past session’s materials and prepare next sessions:

**1**: Yes **2:** No

**Q4.** Your self-evaluation in terms of active participation during the 3 months‘ course. You were:

**1:** Very active **2:** Rather active **3:** Not active, too difficult

**Q5:** How was the course atmosphere (among the participants):

**1:** Friendly/cooperative **2:** OK **3:** Not friendly

The quality of the scientific talks and practical sessions during the course:

Please try to reflect as accurately as possible your opinion about the different parts of the course program:

**Q6.** Updates in Unix/Perl in the context of genome analysis (September 18 – 29)

**Q6.1.** Talks:

**1**:Excellent **2**:Very Good **3**:Good **4**:Adequate **5**:Poor **6**:Unsatisfactory

**Q6.2.** Practical sessions:

**1**:Excellent **2**:Very Good **3**:Good **4**:Adequate **5**:Poor **6**:Unsatisfactory

**Q7.** Sequence Analysis update (October 2 – October 13)

**Q7.1.** Talks:

**1**:Excellent **2**:Very Good **3**:Good **4**:Adequate **5**:Poor **6**:Unsatisfactory

**Q7.2.** Practical sessions:

**1**:Excellent **2**:Very Good **3**:Good **4**:Adequate **5**:Poor **6**:Unsatisfactory

Genome Analyses:

**Q8.** Complete Genomes (October 16 – October 25 and November 6 – 9)

**Q8.1.** Talks:

**1**: Excellent **2**:Very Good **3**:Good **4**:Adequate **5**:Poor **6**:Unsatisfactory

**Q8.2.** Practical sessions:

**1**: Excellent **2**:Very Good **3**:Good **4**:Adequate **5**:Poor **6**:Unsatisfactory

**Q9.** NGS data analyses (October 26 – 31 and November 1 – 2)

**Q9.1.** Talks:

**1**: Excellent **2**:Very Good **3**:Good **4**:Adequate **5**:Poor **6**:Unsatisfactory

**Q9.2.** Practical sessions:

**1**: Excellent **2**:Very Good **3**:Good **4**:Adequate **5**:Poor **6**:Unsatisfactory

**Q10:** NGS technologies - Algorithms (November 13 – November 16)

**Q10.1.** Talks:

**1**: Excellent **2**:Very Good **3**:Good **4**:Adequate **5**:Poor **6**:Unsatisfactory

**Q10.2.** Practical sessions:

**1**: Excellent **2**:Very Good **3**:Good **4**:Adequate **5**:Poor **6**:Unsatisfactory

**Q11.** Metagenomics (November 20 – November 24)

**Q11.1.** Talks:

**1**: Excellent **2**:Very Good **3**:Good **4**:Adequate **5**:Poor **6**:Unsatisfactory

**Q11.2.** Practical sessions:

**1**: Excellent **2**:Very Good **3**:Good **4**:Adequate **5**:Poor **6**:Unsatisfactory

**Q12.** Complete Bacterial genomes (November 27 – November 30)

**Q12.1.** Talks:

**1**: Excellent **2**:Very Good **3**:Good **4**:Adequate **5**:Poor **6**:Unsatisfactory

**Q12.2.** Practical sessions:

**1**: Excellent **2**:Very Good **3**:Good **4**:Adequate **5**:Poor **6**:Unsatisfactory

**Q13.** Lectures (December 4 – December 8): Bioinformatics and Genomes studies: What did we learn and perspectives

**1**: Excellent **2**:Very Good **3**:Good **4**:Adequate **5**:Poor **6**:Unsatisfactory

Lab Meetings: How did you appreciate the Friday Lab Meeting activities?

**Q14:** How do you appreciate, signing for TOCs of scientific journals to follow the scientific publications:

**1**: Excellent **2**: Very useful **3**:useful **4**:Adequate **5**:Not useful

**Q15:** How do you appreciate, the preparation of the projects (read and synthesize bibliographic resources):

**1**: Excellent **2**: Very useful **3**:useful **4**:Adequate **5**:Not useful

**Q16:** How do you appreciate, the project presentation (PowerPoint and talk):

**1**: Excellent **2**: Very useful **3**:useful **4**:Adequate **5**:Not useful

**Q17:** How do you appreciate, the discussion during the Lab Meeting:

**1**: Excellent **2**: Very useful **3**:useful **4**:Adequate **5**:Not useful

**Q18:** Would you suggest to set up similar Lab meetings in your Lab:

**1**: Yes, very useful **2**:No, too much work

**Q19.** The organizational aspects of the course (communication/coffee-breaks/environment/computers/Internet):

**1**: Excellent **2**: Very Good **3**:Good **4**:Adequate **5**:Poor **6**:Unsatisfactory

**Q20.** The whole course program

**1**: Excellent **2:** Very Good **3**:Good **4**:Adequate **5**:Poor **6**:Unsatisfactory

**Q21.** Overall evaluation of the 3 months ‘course

**1**: Excellent **2**: Very Good **3**:Good **4**:Adequate **5**:Poor **6**:Unsatisfactory

**Q22.** Would you suggest future set up of this course?

**1**: Yes, many colleagues need such a course **2:** No, not enough novelty

**Q23.** How much would you suggest to pay for the participation to this course (including course material, USB flash, coffee-breaks, lunches, ..)?

**1**: 1000DT **2:** 2000DT **3**: 3000DT

**Add free comments (200 words maximum):**

Thank you for your cooperation

The organizers
