# Supplementary material for: Designing and running an advanced Bioinformatics and genome analyses course in Tunisia
Source: PLoS Comput Biol. 2019 Jan 28;15(1):e1006373. doi: 10.1371/journal.pcbi.1006373 (PMC6349305; doi:10.1371/journal.pcbi.1006373)
Supplement: S17 Text — This document includes the statistical outputs represented by histograms as obtained from the completed questionnaire (see S16 Text) by all participants. The histograms show the distribution of the evaluations related to each question of the questionnaire (x-axis) following the number of participants (y-axis). On top of each histogram is indicated the corresponding question. The histograms are followed by the list of mentioned free comments. (DOCX) [file pcbi.1006373.s017.docx]

**S17 Text: Statistical results from the completed evaluation questionnaire**

**Overall evaluations by the participants and comments about the 3 months course**

This document includes the statistical outputs represented by histograms as obtained from the completed questionnaire (S16 Text: Evaluation questionnaire) by all participants. The histograms show the distribution of the evaluations related to each question of the questionnaire (x-axis) following the number of participants (y-axis). On top of each histogram is indicated the corresponding question.

The histograms are followed by the list of mentioned free comments.

**Bioinformatics and Genome Analyses Course**

**September 18 – December 15, 2017**

**Institut Pasteur Tunis**

Statistics obtained from the completed questionnaires (see S16 Text) by all participants were analyzed and results are shown on the following histograms.

Each histogram shows the distribution of the evaluations related to each question of the questionnaire (x-axis) following the number of participants (y-axis). Each histogram is preceded by its corresponding question.

As shown on most histograms, the participants mentioned “excellent”, “very good” or “good” evaluations expressing the global success of the course.

Only questions about the Lab meeting organization showed one or 2 participants that were not satisfied because of the extra workload generated by the preparation for the Lab meeting sessions.

The histograms are followed by the list of mentioned free comments. Most of the comments expressed satisfaction about the program topics and organization as well as suggestions of topics that should be introduced in future course organization.

**Overall evaluations of the course announcement, background and self-active participation during the course**

Q1: Were the course program and organization adequately announced in terms of content and effective realization?

Q2: Was your background adequate to participate in this course?

Q3: Did you have the necessary time (average 3h/day) during the course period to efficiently review past session’s materials and prepare next sessions?

Q4: Your self-evaluation in terms of active participation during the 3 months ‘course?

Q5: How was the course atmosphere?

**Evaluation of the quality of the scientific and practical sessions during the course**

Q6: Updates in Unix/Perl in the context of genome analysis?

The quality of the scientific talks and practical sessions during the course

Q7: Sequence Analysis update?

The quality of the scientific talks and practical sessions during the course

Q8: Complete Genomes?

The quality of the scientific talks and practical sessions during the course

Q9: NGS data analyses?

The quality of the scientific talks and practical sessions during the course

Q10: NGS technologies – Algorithms?

The quality of the scientific talks and practical sessions during the course

Q11: Metagenomics?

The quality of the scientific talks and practical sessions during the course

Q12: Complete Bacterial genomes?

The quality of the scientific talks

Q13: Lectures on Bioinformatics and Genomes : what did we learn and perspectives.

**Evaluation of the Lab Meetings**

Q14: How do you appreciate, signing for TOCs of scientific journals to follow the scientific publications?

Lab Meetings

Q15: How do you appreciate, the preparation of the projects (read and synthesize bibliographic resources)?

Lab Meetings

**Q16:** How do you appreciate, the project presentation (PowerPoint and talk)?

Lab Meetings

Q17: How do you appreciate, the discussion during the Lab Meeting?

Q18: Would you suggest to set up similar Lab meetings in your Lab?

**Overall evaluation of the organization and course program**

Q19: The organizational aspects of the course (communication/coffee-breaks/environment /computers/Internet/…)?

Q20: The whole course program?

Q21: Overall evaluation of the 3 months ’course?

Q22: Would you suggest future set up of this course?

Q23: How much would you suggest to pay for the participation to this course (including course material, USB flash, coffee-breaks, lunches, ...)?

**List of the mentioned comments shown in bulk as written by the participants**

• I would like to thank you very much for the tireless efforts that you were/are making to move this BCGAIPT2017 process forward. I feel really lucky to be part of the group despite my PhD Viva and the preparation of my presentation.

For that, I advise students, in the future, to focus on this training without doing anything else.

BCGA2017 course is a clear success story for Tunisian researchers/directors since all the difficulties surely encountered from the first BCGA2017 call to come to this final day.

Overall, I will be all the time grateful to you.

• There was a huge part of the studies related to bacteria and it would be more helpful for me if there were examples related to plants.

The rhythm of the course was very intense.

It helped me to learn how to work during these circumstances but I think it’s very hard to do that during 3 consecutive months.

• The course was very intensive but profitable with broad subjects

Lectures were interesting and easy to follow

Lab meeting and conference sessions were very useful for me

Too much like high school From 9:00-18:00 , hard to find relaxing time to share ideas and think about what was presented so I suggest to make Shorter breaks in order to finish sessions earlier.

I suggest to include poster session the next time

Special thanks to the Organizers

• I’d like to acknowledge organizers, colleagues and Pasteur Institute administration for the hosting. The guest researcher visits and courses were deeply appreciated. This course was a dream that came true thanks to endeavor of many parts.

I deeply encourage the continuity of this course, which is at an international high level, and having a growing need for several fields.

Please let me suggest some comments:

It would be helpful, to choose one or two programs (*Perl*, *Tidyverse*, *Phyton*) to make the practical sessions easier to follow and to keep focus. But, I understood that the practical session on Perl was useful to someway stimulate the logic (to be at the heart) of Bioinformatics.

Should split the course:

-Bioinformatics\Genomics according to levels (beginner\advanced)

-Literature research, oral presentations, revue writing.

Although I don’t have the position to comment on Bioinformatics, I believe that the progress in this area has been achieved thanks to the hard work of many researchers. Hence, regardless of the new technologies are becoming, big thumb to the pioneers of the matter!

Yes, for updates, but we should keep in mind that great has been the past, glorious would be our present and future! Hats off to organizers and guests!

• It was good but we could have done better!

• The whole course was really good and interesting.

Concerning the project, I would like to suggest to integrate a practical part.

It would have been more useful to get some data from the internet and work on these data.

It would have been also very useful to integrate a functional annotation part to the course.

• I hope a new set up of this course in the future for scientific community because this course is important to ameliorate the level of Tunisian research.

• I would like to thank all the organizers for this course that I found very interesting and useful. It was really a pleasure to know you and a great opportunity for learning and sharing knowledge.

I was very satisfied with the organization of the course and the various topics covered. Also, I was impressed by the efforts the organizers made to make this course successful, especially Mr Tekaia and Mr Boudabous.

Despite the stress that we faced, the atmosphere was great and friendly. However, I’ve few points to make that I wish they’ll be useful for next courses.

First, some topics were not covered in practical sessions, especially detection of structural variants and point mutations. More emphasis needs to be placed on genome analysis in the biological context during the practical sessions.

Finally, there should be a more interactive discussion during some courses to ensure a greater involvement from students, in particular after 4pm.

• I want to thank each one of the organizers for their hard involvement during the course.

• My suggestions for next courses are:

For Lab meeting: Preparing a practical project: Writing a useful script, Creating a WGS pipeline or a Circos.

For the Workshop: Adding functional annotation analysis, Genome Structural variation.

• I learned a lot during this training that I find very useful for my next research. However, I very much preferred that the project was restricted to a practical and non-synthetic work possibly with a small reminder of bibliography and to concentrate in the lab meeting on discussion about the selected TOC and research actuality instead of making projects presentations (despite the fact that I found the presentation projects part and the TOC are very useful).

Finally, I would like to thank all the organizers specially:

 Mr Fredj Tekaia for his patience and his efforts expended during the period of training.

Mr Abbdellatif Boudabbous for his perseverance, without him, this training will not have taken place. Mme Fatma Guerfali and Mr Dhafer, for their listening, availability to offer the best conditions for the smooth running of this course. Mr Hechmi Louzir for the local that he offered us and the end of class dinner that I personally enjoyed. Thank you

To Houssem and Ousema for all the support and availability.
