# Supplementary material for: Designing and running an advanced Bioinformatics and genome analyses course in Tunisia
Source: PLoS Comput Biol. 2019 Jan 28;15(1):e1006373. doi: 10.1371/journal.pcbi.1006373 (PMC6349305; doi:10.1371/journal.pcbi.1006373)
Supplement: S18 Text — This document includes the statistical output represented by a histogram, of the participants evaluation scores by 5 speakers who participated by at least one week lectures and practical sessions. The histogram shows the distribution of the obtained mean-scores (x-axis) following the number of participants (y-axis). The detailed course program and related lectures, documents for practical sessions are also available for public access through the link: https://webext.pasteur.fr/tekaia/BCGAIPT2017/BCGAIPT2017_Prog.html and the GitHub platform: https://github.com/tekaia/BCGAIPT2017. (DOCX) [file pcbi.1006373.s018.docx]

**S18 Text: Overall evaluations of participants by tutors**

This document includes the statistical output represented by a histogram, of the participants evaluation scores by 5 speakers who participated by at least one week lectures and practical sessions. The histogram shows the distribution of the obtained mean-scores (x-axis) following the number of participants (y-axis).

**Bioinformatics and Genome Analyses Course**

**September 18 – December 15, 2017**

**Institut Pasteur Tunis**

Five speakers that participated by at least one week lectures and practical sessions evaluated at the end of their respective sessions each participant with a score of 1 (not enough appropriate for the topic) to 5 (very good) that reflected the awareness and mastering of the delivered topics as well as their active participation during their corresponding lectures and practical sessions (commenting, asking questions, suggesting solutions,…).

The scores were coded as follows:

1-Not enough appropriate for the topic

2-No specific mention

3-Pretty good

4-Good

5-Very good

Mean scores were calculated for each participant. The histogram shows the distribution of the obtained mean-scores (x-axis) following the number of participants (y-axis). The majority of the participants are scored “good” or “very good”.
